# Replication of a synthetic oligomer using chameleon base-pairs†

**DOI:** 10.1039/d2cc04580j

**Published:** 2022-09-12

**Authors:** Diego Núñez-Villanueva, Christopher A. Hunter

**Affiliations:** Yusuf Hamied Department of Chemistry, University of Cambridge Lensfield Road Cambridge CB2 1EW UK herchelsmith.orgchem@ch.cam.ac.uk

## Abstract

Salt bridges were used to attach polymerisable amidine monomers to an oligomeric benzoic acid template. CuAAC oligomerisation reactions in the presence of a benzoic acid 3-mer template gave the amidine 3-mer copy as the major product. Cleavage of ester linkers was used to hydrolyse off the amidine recognition units and convert the product into a benzoic acid 3-mer copy of the original template.

## Abstract 2

An oligomer equipped with benzoic acid recognition units templates the polymerization of monomers with amidine recognition units, but cleavage of ester linkers in the resulting copy transforms the amidine recognition units into benzoic acids.

## Graphical abstract

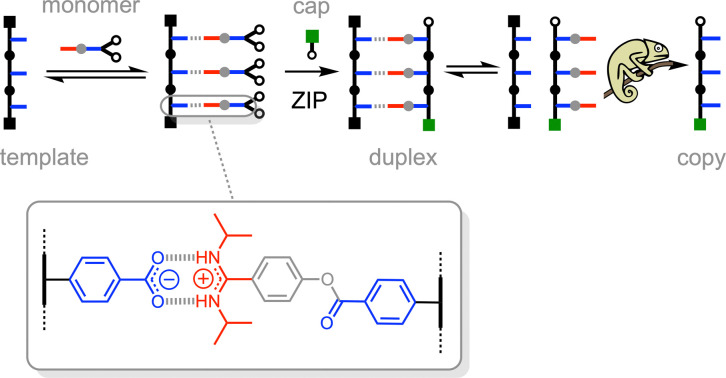

The replication and mutation of linear polymers is the basis for molecular evolution in biological systems. All current technologies for exploiting molecular evolution are based on nucleic acid replication and mutation and are therefore limited to nucleic acids and proteins.^1–7^ To apply molecular evolution principles to synthetic information-containing polymers, new methods for replicating synthetic polymers are required.^8^

We have recently reported a method for sequence information transfer between synthetic oligomers using kinetically inert covalent base-pairing.^9^Fig. 1 illustrates the approach applied to direct replication of a 3-mer template.^10^ In the first step, benzoic acid monomers equipped with a hydroquinone linker, an alkyne and an azide are loaded onto the template using ester coupling reactions to give the pre-ZIP intermediate. In the ZIP step, intramolecular copper catalyzed azide alkyne cycloaddition (CuAAC) reactions lead to oligomerization of the monomers on the template to give the duplex. A chain capping azide is used to block any undesired macrocyclization and intermolecular reactions.^11,12^ Cleavage of the ester bonds in the duplex releases the hydroquinone linkers to regenerate the original template along with the copy strand. We have shown that the nature of the chemical information that is transferred in this replication process can be modulated by varying the chemical connectivity in the base-pair.^13,14^

**Fig. 1:**
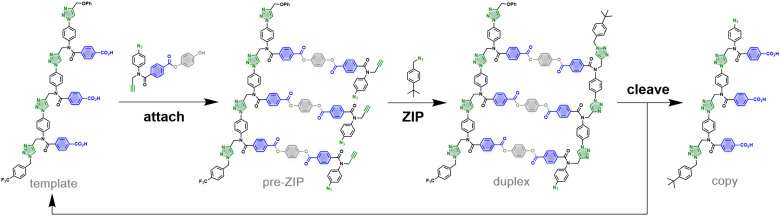
Replication of a 3-mer template using covalent base-pairs with traceless linkers. Monomers are loaded onto the template *via* ester coupling. The resulting pre-ZIP intermediate is subjected to a CuAAC reaction in the presence of 4-*tert*-butylbenzyl azide as a chain capping agent to obtain the duplex. Hydrolysis of the ester base-pairs in the cleave step regenerates the template together with the copy strand.

Here, we introduce a non-covalent version of the approach outlined in Fig. 1. Fig. 2 shows a similar replication process, but where one of the ester linkages in the base-pair has been replaced by a salt bridge between an amidine and a benzoic acid. Self-assembly of the pre-ZIP intermediate can be achieved by simply mixing the 3-mer template with benzoic acid monomers equipped with an amidine, an alkyne and an azide. The amidine-benzoic acid interaction should be strong enough to ensure quantitative loading of monomers onto the template in non-polar solvents.^15–23^ In the CuAAC ZIP step, intramolecular reactions on the template should be favoured relative to off-template intermolecular processes, leading to the salt bridge duplex. Hydrolysis of the ester linkers will cleave off the amidine recognition units, destroy the duplex, and release the original template along with the benzoic acid 3-mer copy.

**Fig. 2:**
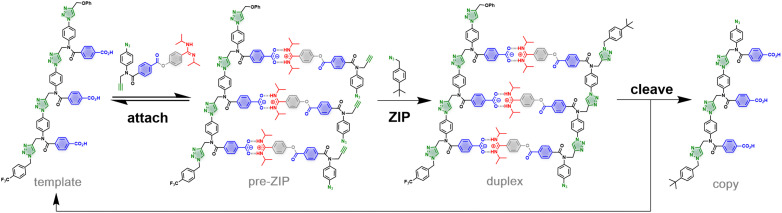
Replication of a 3-mer template using salt bridge base-pairs with cleavable linkers. Mixing the template with the monomers leads to self-assembly of the pre-ZIP intermediate due to salt bridge formation. CuAAC oligomerization in the presence of the 4-*tert*-butylbenzyl azide capping agent leads to the non-covalent duplex. Hydrolysis of the esters in the cleave step removes the amidines to regenerate the template and release the copy strand.

The chameleon base-pairing system illustrated in Fig. 2 provides a useful strategy for switching the identities of the recognition units on an oligomer. Monomers are presented to the template as amidines, so that they are bound by the complementary benzoic acid recognition units. The initial copy strand formed in the replication process is an oligoamidine, but hydrolysis of the ester linkers transforms the amidine recognition units into benzoic acid recognition units. This chameleon-like switching of the recognition units on the copy strand means that it will be able to base-pair with the same amidine monomers as the original template.

This communication describes an implementation of this strategy for the replication of a benzoic acid 3-mer. The efficiency of the templating process is established by showing that very different product distributions are obtained from oligomerization reactions carried out in the presence and absence of a template. In the untemplated oligomerization reaction, a statistical mixture of oligomers of different chain lengths is obtained (Fig. 3), and the average chain length is determined by the amount of chain capping agent added to the CuAAC reaction mixture. In the templated reaction, a single chain length, which is dictated by the length of the template, predominates.

**Fig. 3:**
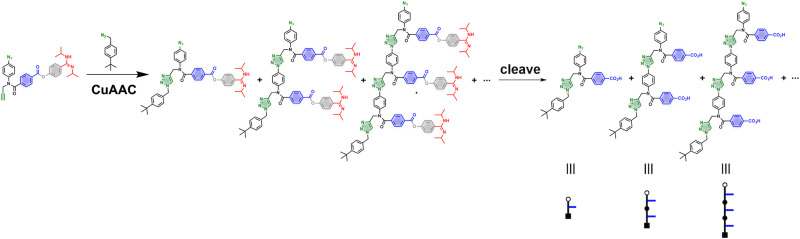
Non-templated oligomerization of the monomer in the presence of the 4-*tert*-butylbenzyl azide capping agent leads to a distribution of oligomers of different lengths. The cleave step hydrolyses the esters and generates the corresponding carboxylic acid oligomers.

We first carried out the synthesis of the substrates needed for the experiments highlighted in Fig. 2 and 3 (Scheme 1). The 3-mer template has been previously reported as well as benzoic acid monomer 5.^1,10^ To prepare the amidine monomer, 4-iodophenol 1 was quantitatively protected with TBDPS-Cl to give 2, which was subsequently lithiated and then treated with *N*,*N*′-diisopropylcarbodiimide (DIC) affording amidine 3 in good yield. After removal of the silyl protecting group, phenol 4 was coupled with 5 using EDC. The product of this reaction was initially obtained as a salt after chromatography, but washing with aqueous NaHCO_3_ gave amidine 6 in excellent yield.

**Scheme 1:**
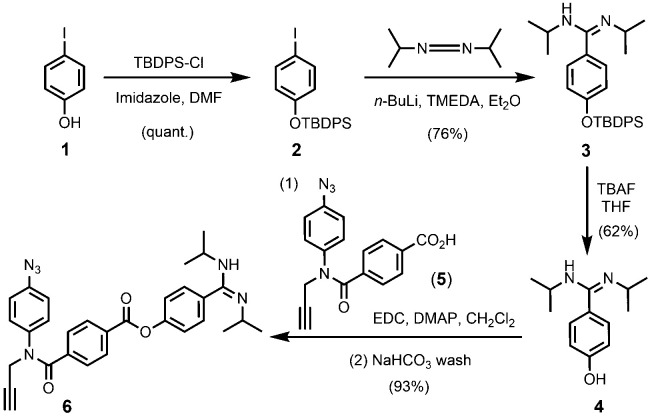


^1^H NMR titrations were carried out in deuterochloroform to assess the strength of the amidine-benzoic acid interaction (see ESI† for details). Addition of benzoic acid to 6 leads to significant changes in the chemical shifts of the signals due to the amidine NH and isopropyl groups, which are indicative of salt bridge formation. The titration data show that a 1 : 1 complex was formed, and although the association constant was too large to be accurately determined, we can place a lower limit of 10^4^ M^−1^ on the value, which is consistent with literature reports.^17–22^

An initial optimization of the CuAAC reaction conditions was carried out exploring a range of substrate concentrations, solvents and capping azide concentrations (see ESI† for details). Fig. 4 shows results obtained working at 0.1 mM of template, 0.3 mM of the monomer and 1 mM of capping azide in dichloromethane. The UPLC traces correspond to the crude reaction mixtures obtained after the complete replication cycle shown in Fig. 2, *i.e.* CuAAC reaction followed by hydrolysis. In the absence of the template, a distribution of oligomers is obtained, with the 1-mer and 2-mer as major species. The reason that only short oligomers were observed in this experiment is that the capping azide is present in a large excess, so reaction of the monomer with the capping azide is the most probable process. When the 3-mer template was present, the yield of 2-mer decreased dramatically, and there was a clear amplification of the length-complementary 3-mer product. Note that it is possible to distinguish the template from the copy strand in the UPLC trace, because they have different end groups.

**Fig. 4:**
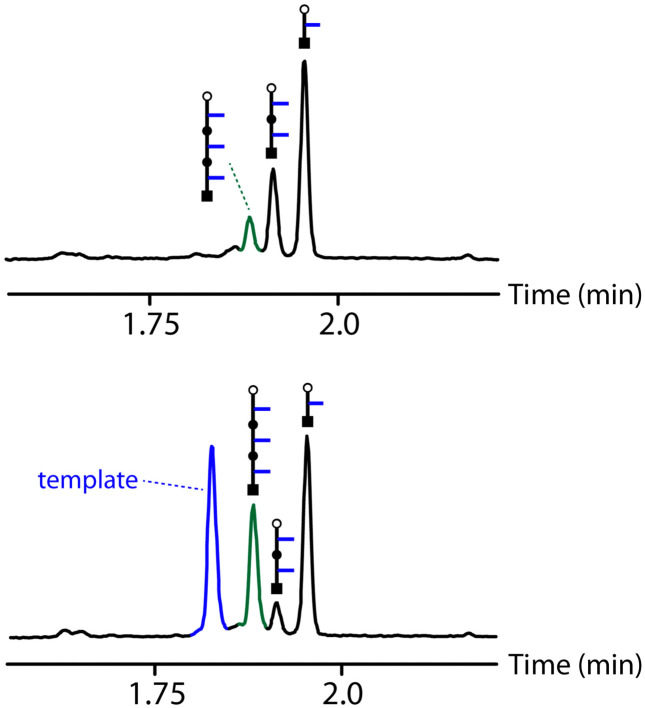
UPLC traces of the crude reaction mixtures obtained from the replication cycle shown in Fig. 2 (see Fig. 3 for the chemical structures of the oligomers indicated by cartoons). Oligomerization of 6 was carried out in the absence (top) and presence (bottom) of template: 0.3 mM 6, 0.1 mM template, 0.6 mM Cu(CH_3_CN)_4_PF_6_, 0.6 mM TBTA, 1 mM 4-*tert*-butylbenzylazide in CH_2_Cl_2_ at r.t. for 48 h, followed by 1 M LiOH in THF : H_2_0 3 : 1 for 1 h. UPLC method: C18 column at 40 °C (254 nm) using water + 0.1% formic acid (A) and CH_3_CN + 0.1% formic acid (B); Gradient of 0–2 min 5–100% B + 1 min 100% B.

Fig. 5 shows the different reaction pathways that are possible for the templated and untemplated reactions. In the untemplated process all reactions are intermolecular, so a range of different chain lengths are obtained. The capping agent is a benzyl azide, so the rate of reaction with this species (*k*′) is larger than the rate of reaction with the aryl azide present in the monomer (*k*).^11^ In addition, the capping agent is present in excess, so the major product in the untemplated process comes from reaction of the monomer with the chain capping azide. In the presence of a template, any unbound monomers will react *via* the same pathway as the untemplated reaction. However, the rate of reaction between two monomers that are bound to the template will be enhanced by the kinetic effective molarity for this intramolecular process (EM^†^). Any 2-mer that is formed by an off-template intermolecular reaction will be bound to the template with a much higher affinity than the monomer (Fig. 5). As a result, 2-mers are much more likely to be trapped by an intramolecular templated reaction than monomers, which explains the very low yield of 2-mer observed in the presence of template in Fig. 4. The efficiency of the templating process is therefore determined by the association constants for binding to the template (*K*_1_ and *K*_2_ in Fig. 5) and EM^†^ for the templated reaction.

**Fig. 5:**
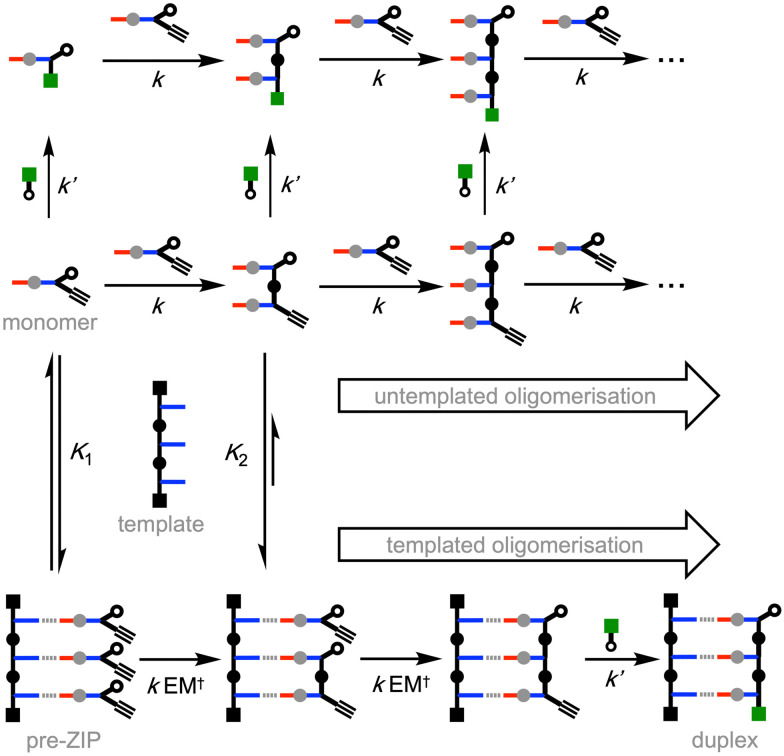
Different reaction pathways for the CuAAC oligomerization of an alkyne-azide monomer in the presence and absence of a complementary 3-mer template. In the untemplated pathway (top channel), all reactions are intermolecular processes, and the product distribution is governed by the reaction rate constants (*k* and *k*′) and the concentrations of the monomer and chain capping agent. In the templated pathway (bottom channel), intramolecular reactions between monomers bound to the template are favoured by the kinetic effective molarity (EM^†^). If the association constants (*K*_1_ and *K*_2_) and the EM^†^ are sufficiently high, the templated duplex will be the major product.

Intermolecular reactions with the capping agent can compete with intramolecular reactions on the template, and this process can be used to estimate the value of EM^†^ for the templated reaction. By measuring the relative amounts of 2-mer and 3-mer obtained in the presence of template and increasing concentrations of capping agent, a value of 32 mM was obtained for EM^†^ (see ESI† for details). The value of EM^†^ is an order of magnitude higher than the concentration of monomer used in these experiments (0.3 mM), and so templated replication is faster than off-template oligomerisation. However, the value of EM^†^ is an order of magnitude lower than the corresponding value measured using the covalent base-pairing system shown in Fig. 1.^11^ This result suggests that either the salt bridge is less well-organised than the covalent system or that there is a difference in geometry that reduces the reaction rate.

Molecular modelling was used to investigate the difference between the covalent and non-covalent templating processes. The ring strain associated with the products of the templated oligomerisation reactions shown in Fig. 1 and 2 was calculated (see ESI† for details). For both systems, there is no significant ring strain (20–30 kJ·mol^−1^ per macrocyclization), and there is no obvious difference between the two architectures.

In conclusion, we have demonstrated replication of a synthetic triazole oligomer using a combination of salt bridge base-pairs and a cleavable linker. The amidine-carboxylate interaction is strong enough for efficient assembly of the monomer units on the template under the dilute conditions required to suppress off-template reactions. Oligomerisation using CuAAC reactions in the presence of the 3-mer benzoic acid template selectively amplified the formation of the length complementary 3-mer benzoic acid copy. In contrast, in the absence of template a mixture of different chain lengths was obtained. A key feature of the salt bridge base-pairing system described here is that the amidine recognition unit is attached to the monomer *via* an ester linker. Cleavage of these ester linkers after replication can be used to convert an amidine oligomer into a benzoic acid oligomer, thereby switching the identity of the recognition units. The cleavable linker chameleon base-pair approach offers a general strategy for manipulating recognition sites on synthetic oligomers to control template-directed sequence information transfer processes.

The manuscript was written through contributions of all authors. We thank the Engineering and Physical Sciences Research Council (EP/P027067/1), the European Research Council (ERC-2020-AdG101018984-InfoMols) and the Herchel Smith Fund for funding.

## Conflicts of interest

There are no conflicts to declare.

## Supplementary Material

CC-058-D2CC04580J-s001
