# Supplementary material for: Replication of a synthetic oligomer using chameleon base-pairs
Source: Chem Commun (Camb). 2022 Sep 12;58(78):11005–8. doi: 10.1039/d2cc04580j (PMC9521413; doi:10.1039/d2cc04580j)
Supplement: CC-058-D2CC04580J-s001 [file CC-058-D2CC04580J-s001.pdf]

Electronic Supplementary Material (ESI) for ChemComm.
This journal is © The Royal Society of Chemistry 2022

Supporting information

Replication of a synthetic oligomer using chameleon base-pairs

Diego Núñez-Villanueva and Christopher A. Hunter*

Yusuf Hamied Department of Chemistry, University of Cambridge, Lensfield Road, Cambridge CB2 1EW, UK. E-mail: herchelsmith.orgchem@ch.cam.ac.uk

TABLE OF CONTENTS **Page**

1. General experimental details. S2

2. Synthesis and characterization of compounds S3

3. Molecular modelling S18

4. Binding studies S21

5. Templating experiments S22

6. References S29

1. General experimental details.

All the reagents and materials used in the synthesis of the compounds described below were bought from commercial sources, without prior purification. Dry THF and CH_2Cl_2 were obtained from a solvent purification system (Pure Solv™, Innovative Technology, Inc.). Anhydrous DMF was purchased from Sigma-Aldrich. Thin layer chromatography was carried out using with silica gel 60F (Merck) on glass plates. Flash chromatography was carried out on an automated system (Combiflash Rf+ or Combiflash Rf Lumen) using prepacked cartridges of silica (25µ PuriFlash® columns). All NMR spectroscopy was carried out on a Bruker 400 MHz DPX400, 400 MHz AVIII400, 500 MHz DCH cryoprobe or 500 MHz TCI Cryoprobe spectrometer using the residual solvent as the internal standard. All chemical shifts (δ) are quoted in ppm and coupling constants given in Hz. Splitting patterns are given as follows: s (singlet), bs (broad singlet), d (doublet), t (triplet), q (quadruplet), m (multiplet). FT-IR spectra were measured on a Bruker Alpha spectrometer equipped with an ATR cell. UPLC analysis of samples was performed using Waters Acquity H-class UPLC coupled with a single quadrupole Waters SQD2. Acquity UPLC CSH C18 column, 130 Å, 1.7 µm, 2.1 mm x 50 mm or Acquity UPLC BEH C8 column, 130 Å, 1.7 µm, 2.1 mm x 50 mm were used as UPLC columns. The conditions of the UPLC method are as follows: gradients of water + 0.1% formic acid (solvent A) and acetonitrile + 0.1% formic acid (solvent B) as specified in each case. Flow rate: 0.6 ml/min; Column temperature of 40°C; Injection volume of 2 µL. The signal was monitored at 254 nm. HRMS analysis was performed in an Agilent walk up 6230 LC/TOF using a gradient from 5 to 100% of acetonitrile (0.25% formic acid) in water (0.25% formic acid) over 6 minutes.

Template[S1] and compound **5**[S2] have been previously reported.

2. Synthesis and characterization of compounds.

Synthesis of compound 2.

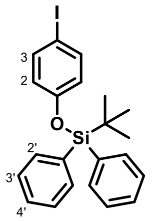

A solution of *p*-iodophenol **1** (2.00 g, 9.09 mmol) in DMF (10 mL) was treated with imidazole (1.24 g, 18.18 mmol) and TBDPS-Cl (2.84 mL, 10.91 mmol). The solution was stirred at room temperature for 15 h. Then, the mixture was acidified with 1% HCl solution to pH = 3 and extracted with EtOAc (3x). The organic layers were joined and washed with 5% LiCl aq. soln. (2x) and brine (1x). The solvent was evaporated to dryness and the residue was purified by flash chromatography on silica gel using pet. ether as solvent to afford **2** (4.17 g, quant.) as a clear oil.

^{1}H NMR (400 MHz, $CDCl_3$): δ_H = 7.70 (d, 4H, *J* = 8.0 Hz, 2'-H, TBDPS), 7.44 (m, 2H, 4'-H, TBDPS), 7.38 (m, 6H, 3-H; 3'-H, TBDPS), 6.54 (d, 2H, *J* = 9.0 Hz, 2-H), 1.10 (s, 9H, CH_3, ^{t}Bu, TBDPS).

^{13}C NMR (100.6 MHz, $CDCl_3$): δ_C = 155.7 (1-C), 138.2 (3-C), 135.6 and 135.6 (2'-C, TBDPS), 132.6 (1'-C, TBDPS), 130.2 (4'-C, TBDPS), 128.0 (3'-C, TBDPS), 122.3 (2-C), 83.6 (4-C), 26.6 (CH_3, ^{t}Bu, TBDPS), 19.6 (C, ^{t}Bu, TBDPS).

HRMS (ES+): calcd for $C_{22}H_{23}IOSi$ 459.0636 $[M+H]^+$, found 459.0620 $[M+H]^+$.

FT-IR (ATR): ν_{max} 2956, 2931, 2858, 1585, 1484, 1271, 1254, 1113, 914 and 823 cm^{-1}.

^{1}H-NMR (400 MHz, $CDCl_3$) compound 2

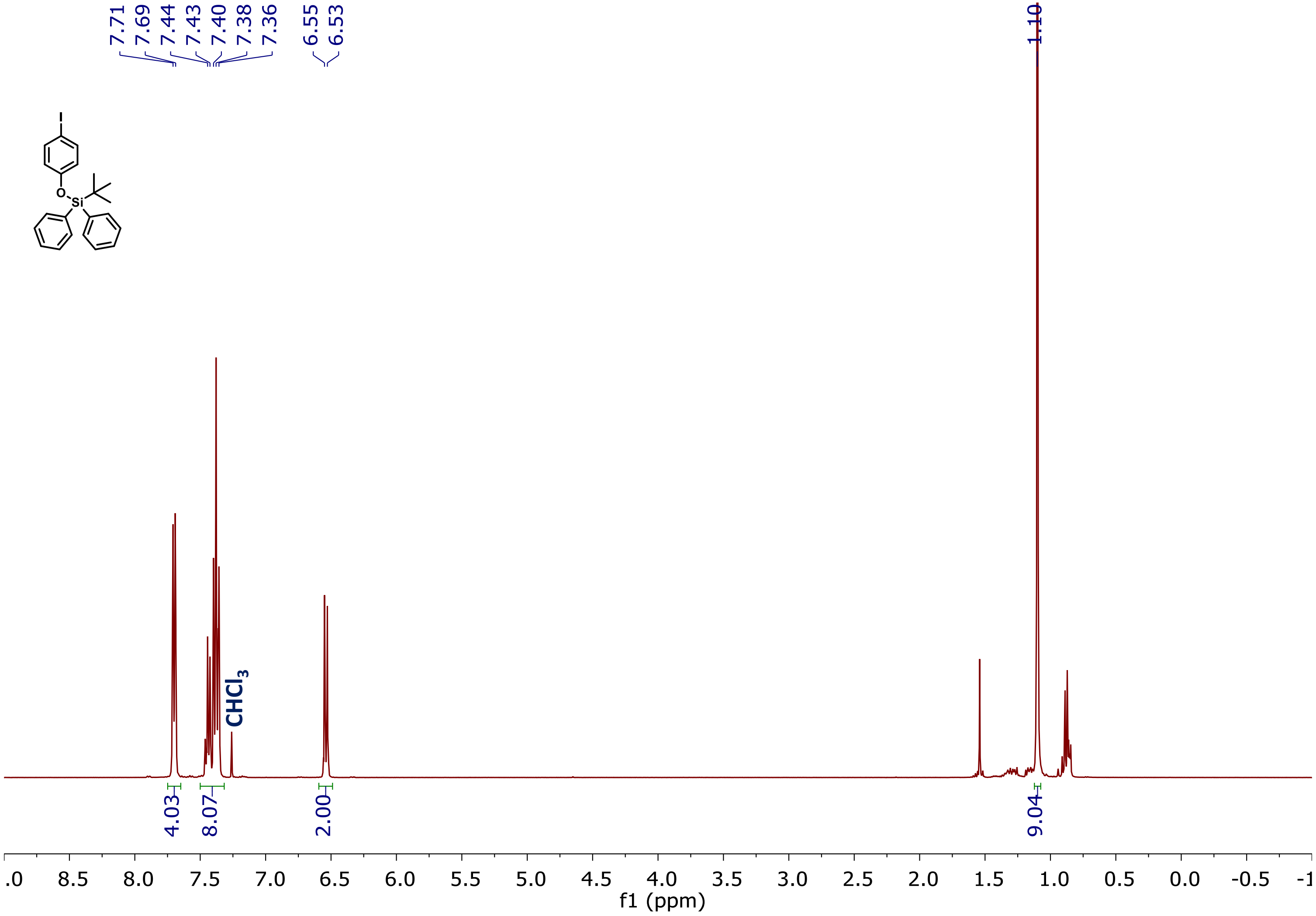

^{13}C-NMR (100.6 MHz, $CDCl_3$) compound 2

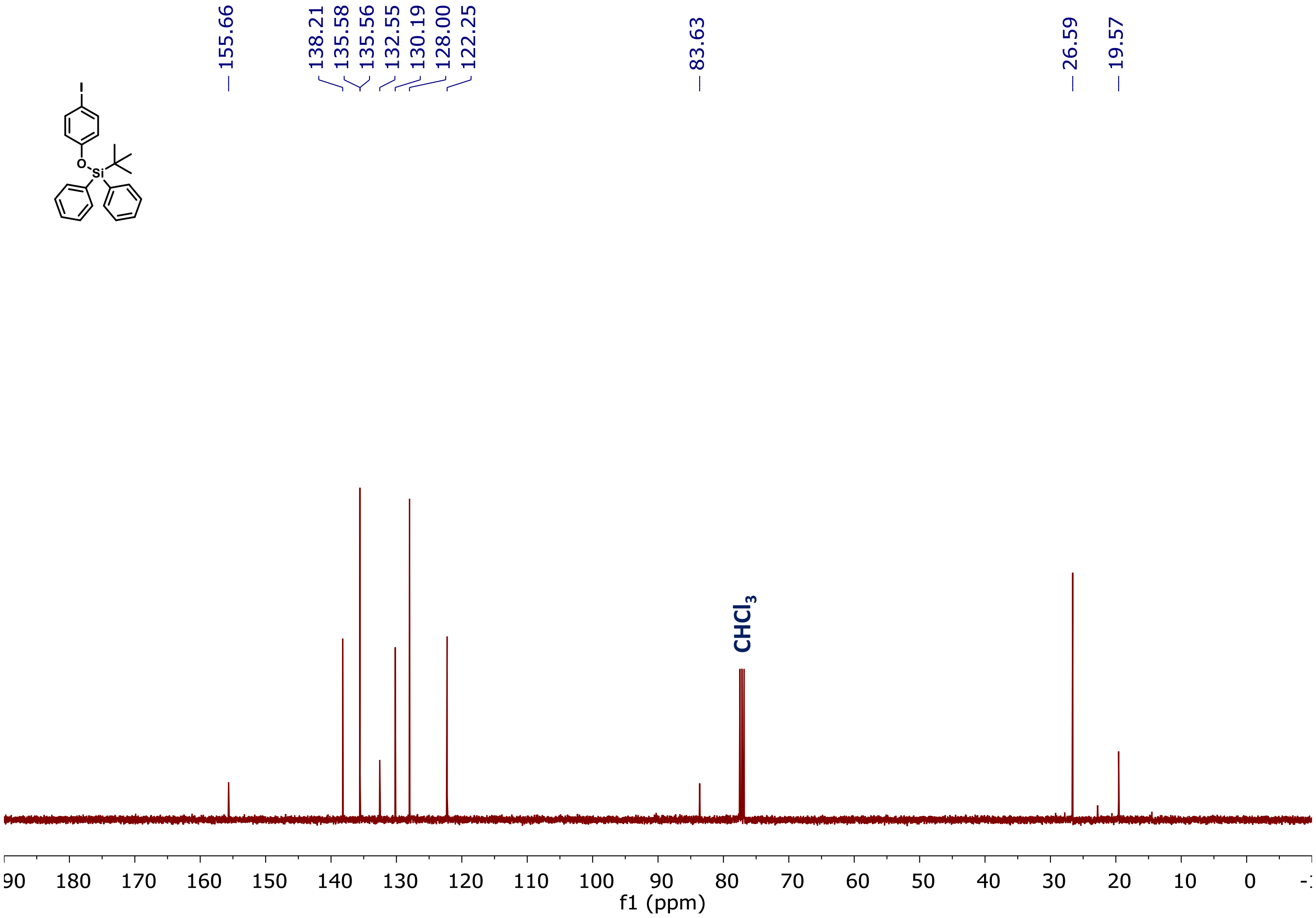

Synthesis of compound 3.

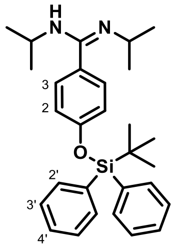

To a solution of **2** (0.300 g, 0.65 mmol) in anhydrous Et_2O (4 mL) at 0 °C, a 1.6 M *n*-hexane solution of *n*-butyllithium (0.450 mL, 0.72 mmol) and *N*,*N*,*N'*,*N'*-tetramethylethylenediamine (TMEDA) (0.107 mL, 0.72 mmol) were added dropwise. After 15 minutes of stirring at 0 °C, a solution of *N*,*N*'-diisopropylcarbodiimide (0.111 mL, 0.72 mmol) in anhydrous Et_2O (1 mL) was added and the resulting mixture stirred at room temperature for 16 h. Then, the solution was poured into water at 0 °C, and extracted with Et_2O (3x). The ether layers were combined and washed with brine (1x), then dried over anhydrous $MgSO_4$ and concentrate under vacuum. The obtained residue was purified by flash chromatography on basic alumina (from 50% to 100% of EtOAc in Pet. Ether) to afford **3** (0.227 g, 76%) as a yellow amorphous solid.

^{1}H NMR (500 MHz, $CDCl_3$): δ_H = 7.71 (d, 4H, *J* = 7.0 Hz, 2'-H, TBDPS), 7.43 (m, 2H, 4'-H, TBDPS), 7.37 (m, 4H, 3'-H, TBDPS), 6.97 (bs, 2H, 3-H), 6.75 (d, 2H, *J* = 8.0 Hz, 2-H), 3.47 (bs, 2H, CH, ^{i}Pr), 1.11 (s, 9H, CH_3, ^{t}Bu, TBDPS), 1.05 (bs, 12H, CH_3, ^{i}Pr).

^{13}C NMR (125.8 MHz, $CDCl_3$): δ_C = 161.5 (C=N, amidine), 155.9 (1-C), 135.7 (2'-C, TBDPS), 132.8 (1'-C, TBDPS), 130.1 (4'-C, TBDPS), 129.7 (4-C), 128.7 (3-C), 127.9 (3'-C, TBDPS), 119.7 (2-C), 46.9 (CH, ^{i}Pr), 26.7 (CH_3, ^{t}Bu, TBDPS), 24.3 (CH_3, ^{i}Pr), 19.6 (C, ^{t}Bu, TBDPS).

HRMS (ES+): calcd for $C_{29}H_{38}N_2OSi$ 459.2826 $[M+H]^+$, found 459.2806 $[M+H]^+$.

FT-IR (ATR): ν_{max} 2961, 2930, 2858, 1626, 1605, 1507, 1463, 1428, 1257, 1111 and 914 cm^{-1}.

^{1}H-NMR (500 MHz, $CDCl_3$) compound 3

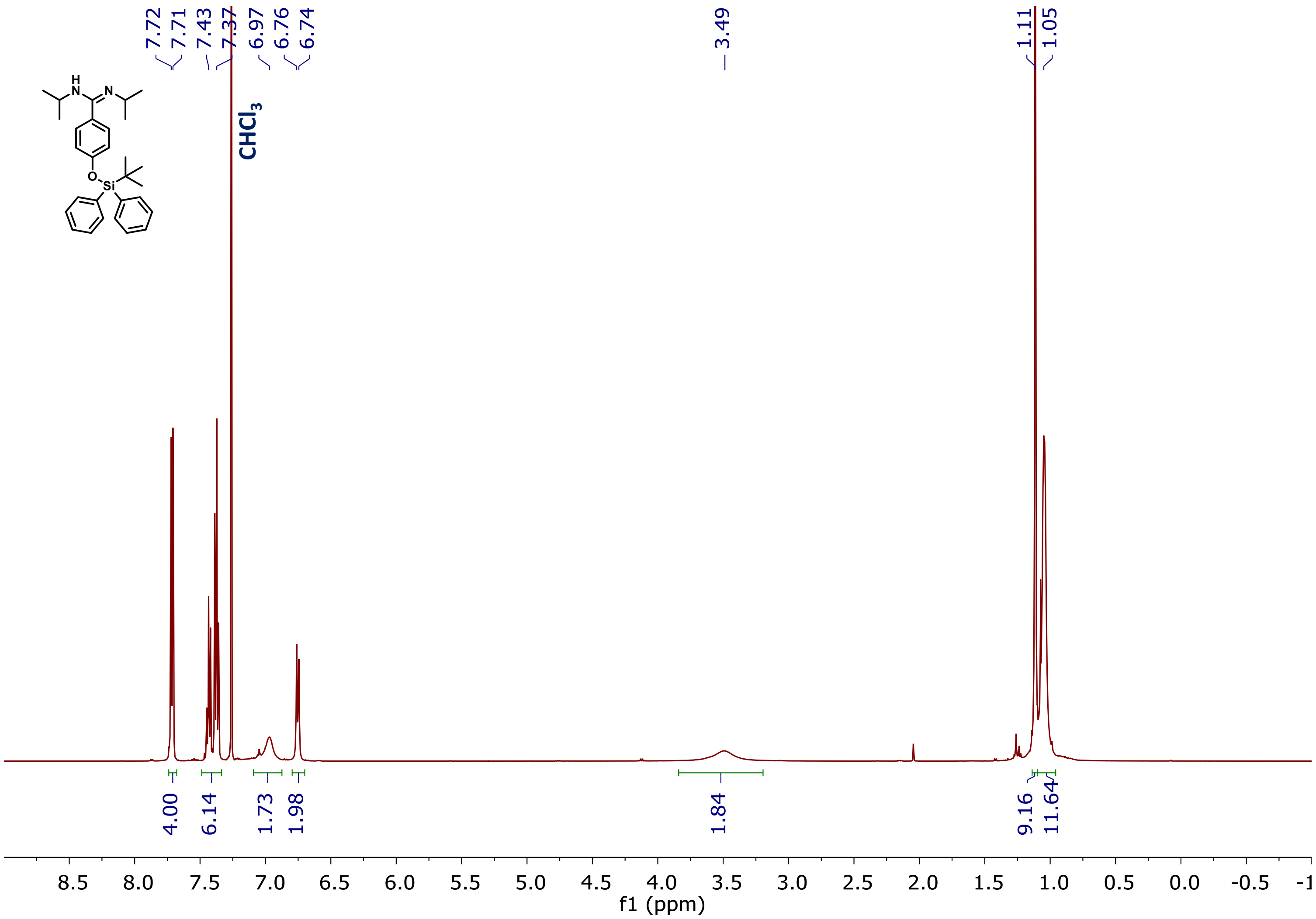

^{13}C-NMR (125.8 MHz, $CDCl_3$) compound 3

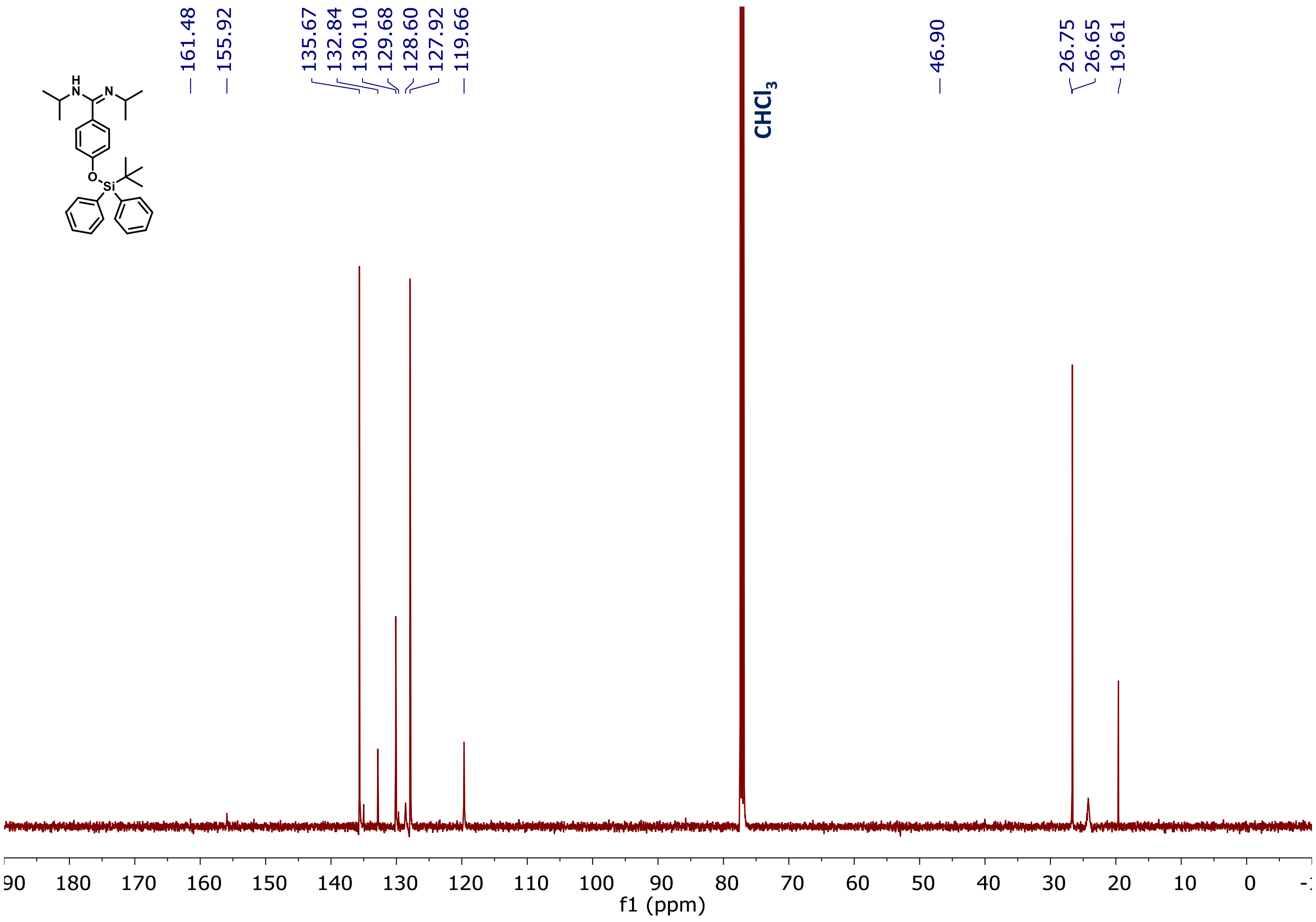

Compound 4.

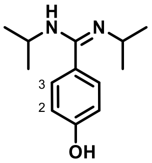

To a solution of **3** (0.096 g, 0.21 mmol) in dry THF (2 mL) under N_2 atmosphere, TBAF (1M in THF, 0.21 mL, 0.21 mmol) was added. After 30 minutes of stirring at room temperature, the reaction was quenched with 0.1N HCl soln. The mixture was evaporated to dryness, suspended in CH_2Cl_2/MeOH 2:1 and filtrated. The obtained residue was purified by flash chromatography on basic alumina via dry loading on celite (from 0% to 25% of MeOH in CH_2Cl_2) to **4** (0.029 g, 62%) as a white solid.

^{1}H NMR (500 MHz, DMSO-*d*$_6$): δ_H = 7.24 (d, 4H, *J* = 7.0 Hz, 3-H), 6.88 (d, 4H, *J* = 7.0 Hz, 2-), 1.75 (bs, 2H, CH, ^{i}Pr), 1.11 (bs, 12H, CH_3, ^{i}Pr).

^{13}C NMR (125.8 MHz, DMSO-*d*$_6$): δ_C = 161.5 (C=N, amidine), 145.0 (1-C), 129.5 (3-C), 115.8 (2-C), 48.2 (CH, ^{i}Pr), 23.0 (CH_3, ^{i}Pr). 4-C cannot be detected due to broadening.

HRMS (ES+): calcd for $C_{13}H_{20}N_2O$ 243.1468 $[M+Na]^+$, found 243.1475 $[M+Na]^+$.

FT-IR (ATR): ν_{max} 2975, 2930, 2879, 1611, 1552, 1514, 1391, 1284, 1131 and 840 cm^{-1}.

^{1}H-NMR (500 MHz, DMSO-d_6) compound 4

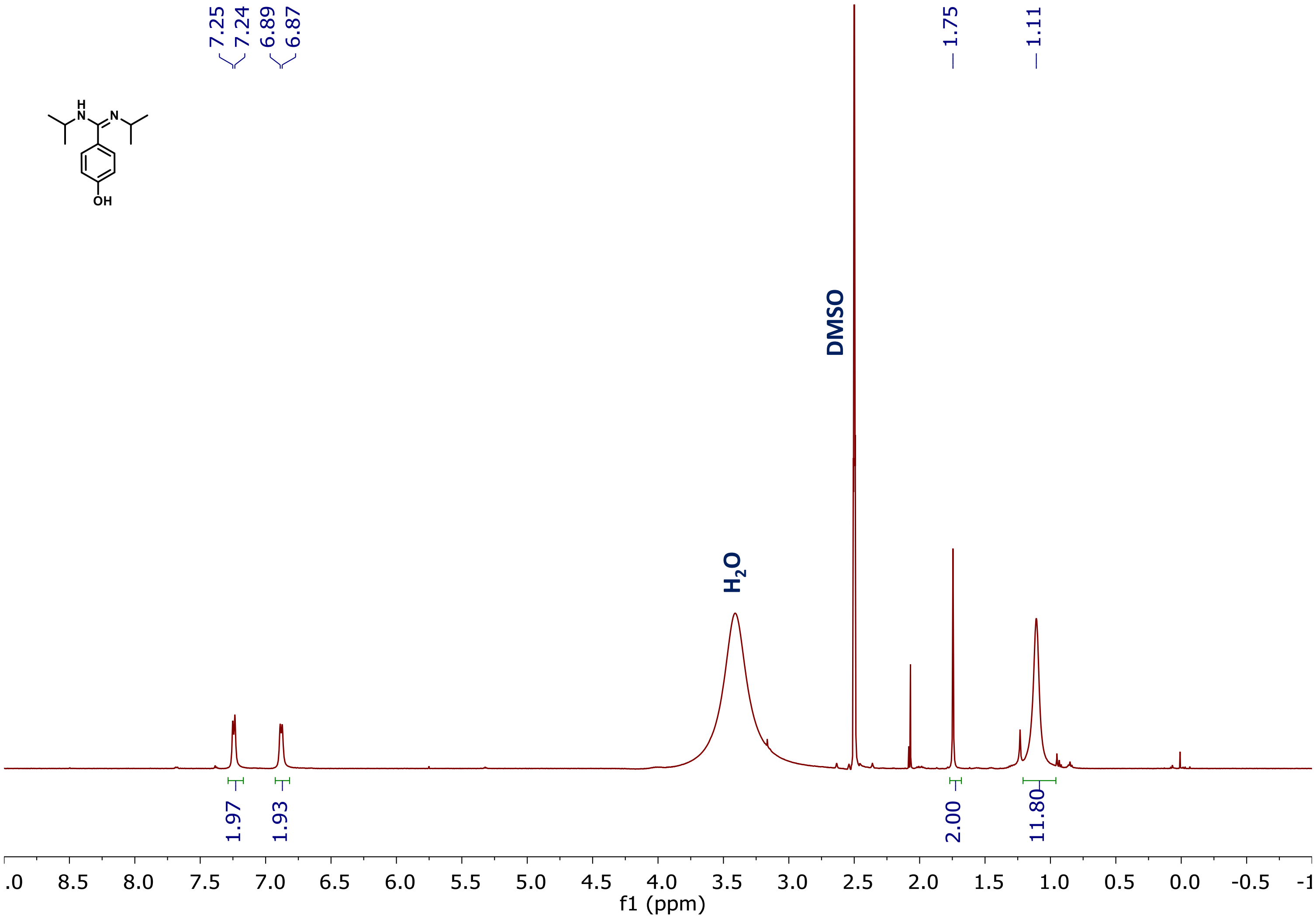

^{13}C-NMR (125.8 MHz, DMSO-d_6) compound 4

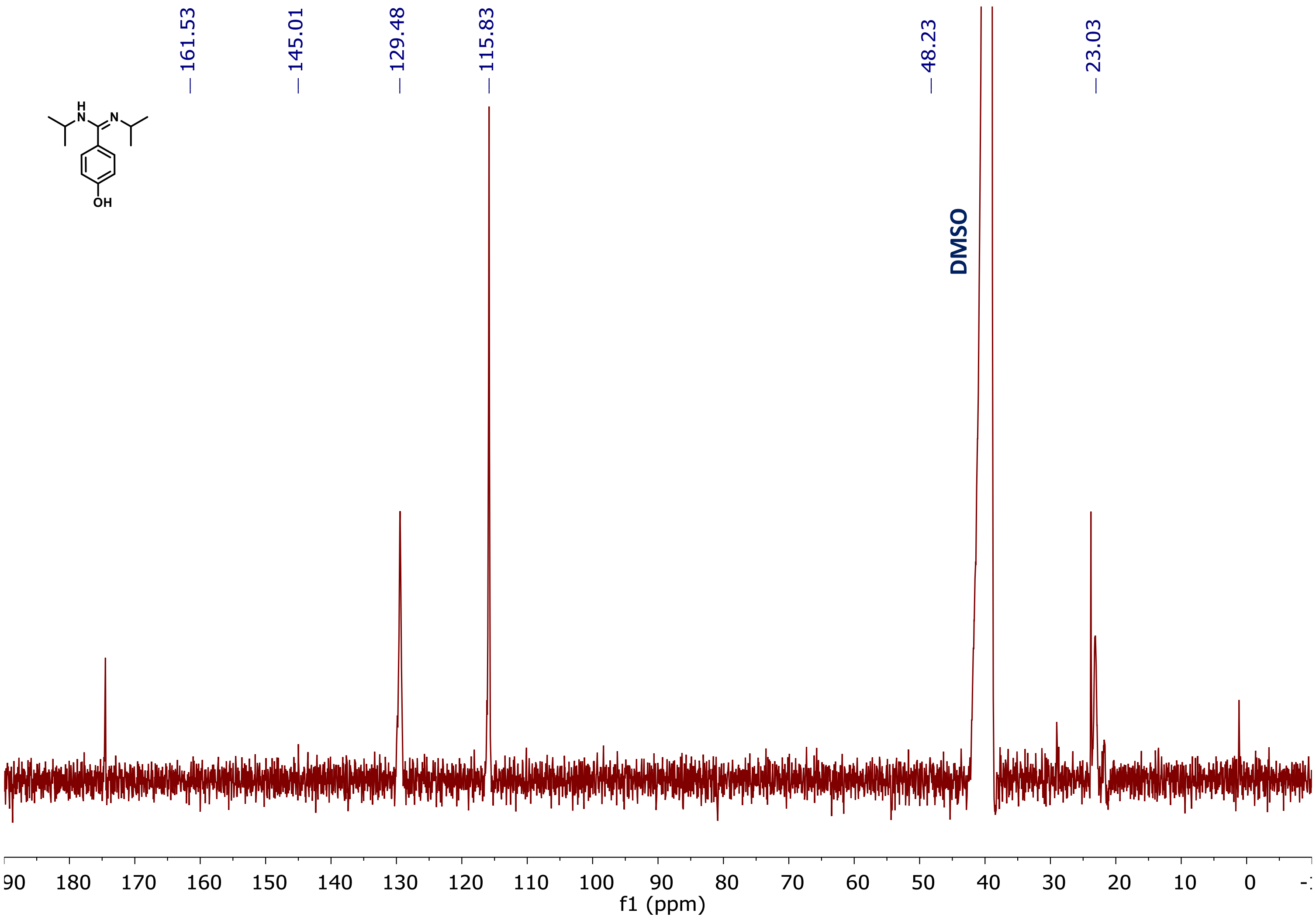

Compound 6·HCl.

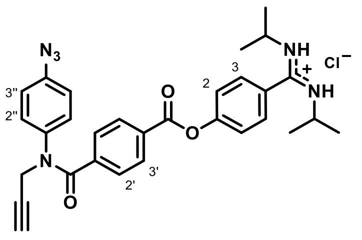

Compound **5**[S2] (0.017 g, 0.05 mmol), compound **4** (0.017 g, 0.08 mmol), EDC (0.061 g, 0.32 mmol) and DMAP (0.019 g, 0.16 mmol) were dissolved in dry CH_2Cl_2 (2.5 mL) under N_2 atmosphere. The reaction was stirred overnight at room temperature. Once finished, the reaction was diluted with EtOAc and washed with 0.1N HCl soln. (2x), H_2O (2x) and brine (1x). The solution was dried with anhydrous $MgSO_4$, filtered and the solvent evaporated. The obtained residue was purified by flash chromatography on silica gel (from 0% to 10% of MeOH in CH_2Cl_2) to afford **6·HCl** (0.023 g, 93%) as a yellow amorphous solid.

^{1}H NMR (500 MHz, CDCl$_3$): δ_H = 10.52 (bs, 2H, NH), 8.04 (d, 2H, *J* = 8.0 Hz, 3'-H), 7.49 (d, 2H, *J* = 8.0 Hz, 2'-H), 7.47 (d, 2H, *J* = 8.5 Hz, 2-H), 7.39 (d, 2H, *J* = 8.5 Hz, 3-H), 7.15 (d, 2H, *J* = 8.5 Hz, 2''-H), 6.95 (d, 2H, *J* = 8.5 Hz, 3''-H), 4.69 (s, 2H, N-CH_2), 3.27 (m, 2H, CH, ^{i}Pr), 2.30 (s, 1H, CH, alkyne), 1.22 (d, 12H, *J* = 6.5 Hz, CH_3, ^{i}Pr).

^{13}C NMR (125.8 MHz, CDCl$_3$): δ_C = 168.9 (CO, amide), 164.3 (C, amidine), 163.8 (CO, ester), 153.4 (1-C), 140.8 (1'-C), 139.8 (4''-C), 138.8 (1''-C), 130.0 (3'-C), 129.8 (4'-C), 129.4 (2''-C), 129.1 (2'-C), 128.3 (3-C), 124.0 (4-C), 123.5 (2-C), 120.1 (3''-C), 78.4 (C, alkyne), 73.1 (CH, alkyne), 47.9 (CH, ^{i}Pr), 39.8 (N-CH_2), 23.4 (CH_3, ^{i}Pr).

HRMS (ES+): calcd for $C_{30}H_{31}N_6ClO_3$ 523.2452 [M-HCl+H]$^+$, found 523.2479 [M-HCl+H]$^+$.

FT-IR (ATR): ν_{max} 2977, 2124, 2093, 1739, 1635, 1505, 1260, 1227, 1166, 1155 and 1066 cm^{-1}.

^{1}H-NMR (500 MHz, $CDCl_3$) compound 6·HCl

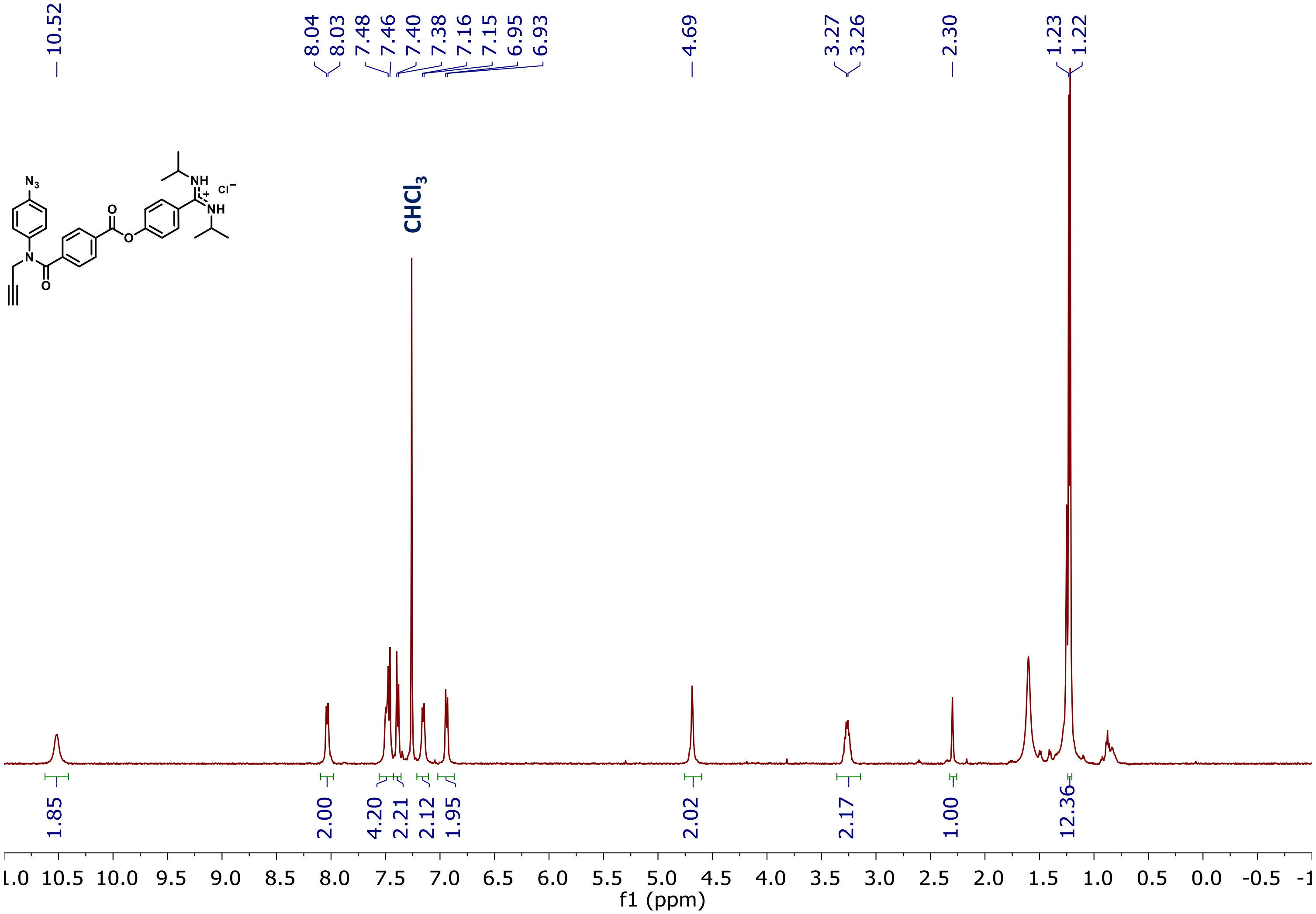

^{13}C-NMR (125.8 MHz, $CDCl_3$) compound 6·HCl

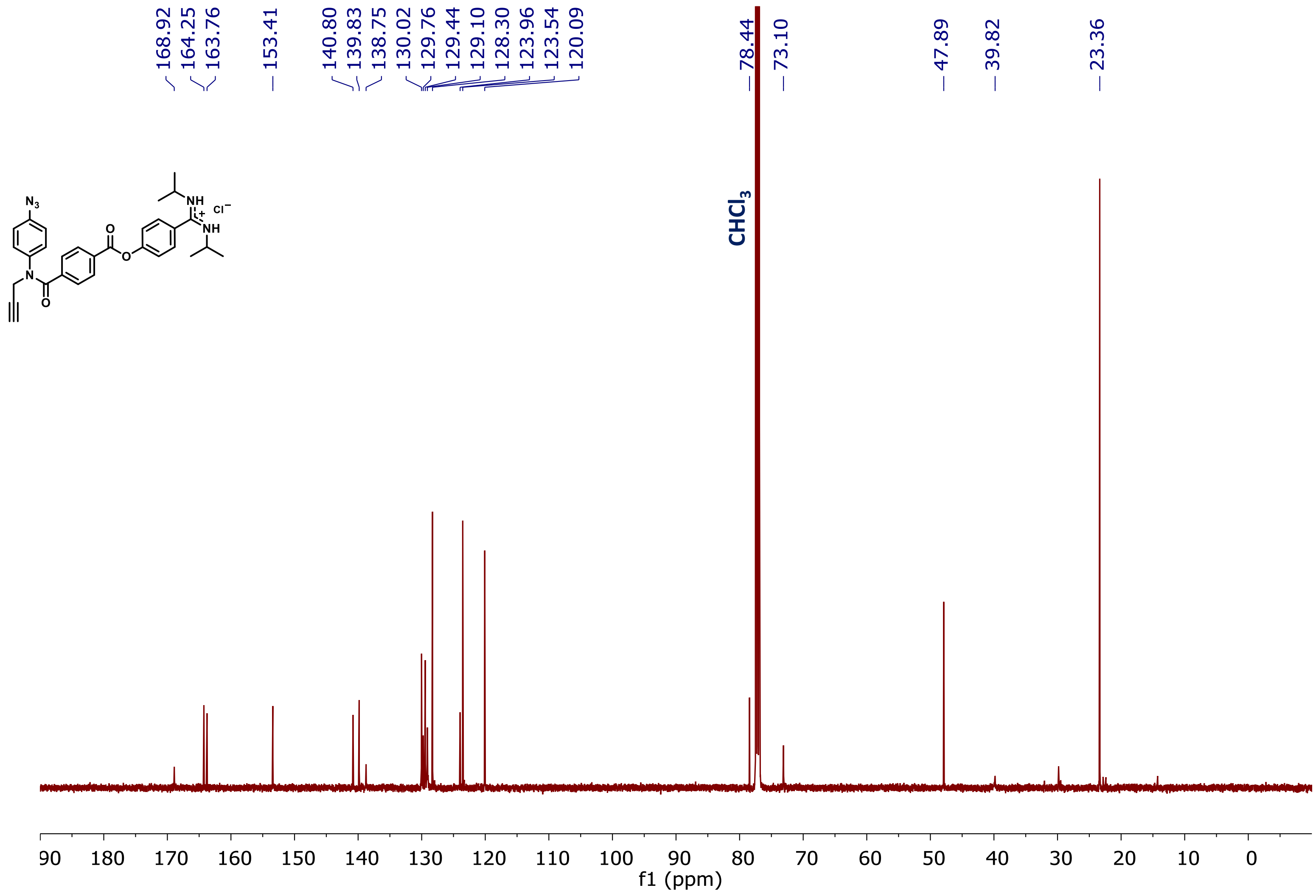

Compound 6.

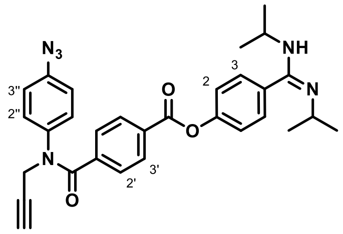

Compound **6·HCl** (0.023 g, 0.04 mmol) was dissolved in EtOAc (10 mL) and then washed with $NaHCO_3$ (3x) and brine. Then, the organic layer was dried over $MgSO_4$ and evaporated to dryness, yielding compound **6** (0.021 g, quant.) as a pale yellow oil.

^{1}H NMR (500 MHz, $CDCl_3$): δ_H = 8.03 (d, 2H, *J* = 8.0 Hz, 3'-H), 7.54 (bs, 1H, NH), 7.47 (d, 2H, *J* = 8.0 Hz, 2'-H), 7.29 (m, 2H, 3-H), 7.24 (m, 2H, 2-H), 7.15 (d, 2H, *J* = 8.5 Hz, 2''-H), 6.94 (d, 2H, *J* = 8.5 Hz, 3''-H), 4.68 (s, 2H, N-CH_2), 3.51 (bs, 2H, CH, ^{i}Pr), 2.29 (t, 1H, *J* = 2.5 Hz, CH, alkyne), 1.11 (bs, 12H, CH_3, ^{i}Pr).

^{13}C NMR (125.8 MHz, $CDCl_3$): δ_C = 169.0 (CO, amide), 163.9 (CO, ester), 153.1 (1-C), 140.7 (1'-C), 139.8 (4''-C), 138.8 (1''-C), 130.0 (3'-C), 129.6 (4-C), 129.4 (2''-C), 129.1 (2'-C and 4'-C), 128.5 (3-C), 123.2 (2-C), 120.1 (3''-C), 78.5 (C, alkyne), 73.1 (CH, alkyne), 47.5 (CH, ^{i}Pr), 39.9 (N-CH_2),23.4 (CH_3, ^{i}Pr); C, amidine was not detected due to broadening.

HRMS (ES+): calcd for $C_{30}H_{30}N_6O_3$ 523.2452 $[M+H]^+$, found 523.2433 $[M +H]^+$.

FT-IR (ATR): ν_{max} 2969, 2927, 2125, 2094, 1739, 1643, 1506, 1294, 1263, 1204 and 1070 cm^{-1}.

^{1}H-NMR (500 MHz, $CDCl_3$) compound 6

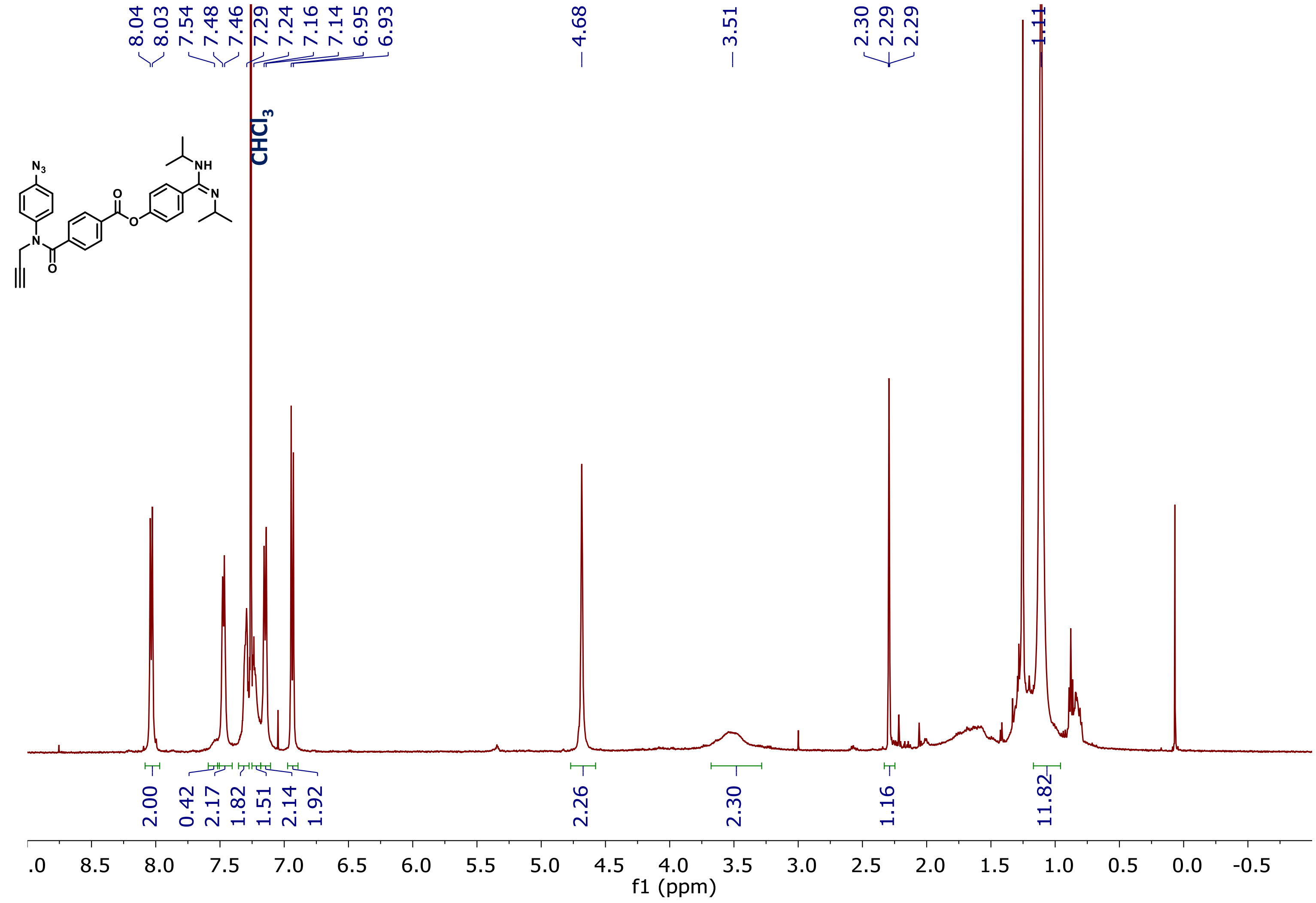

^{13}C-NMR (125.8 MHz, $CDCl_3$) compound 6

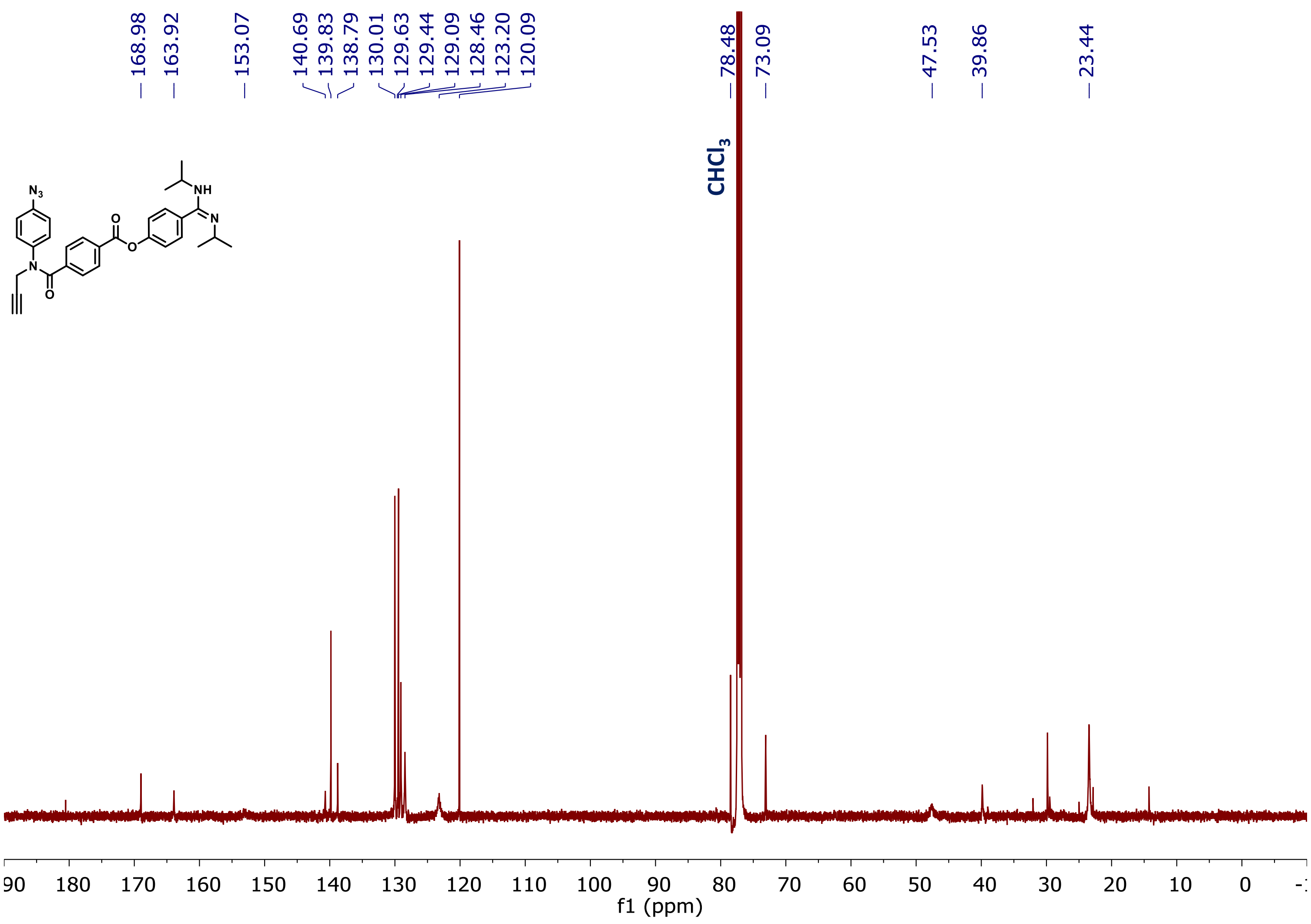

3. Molecular modelling

3.1. General details

Molecular mechanics calculations were performed using MacroModel version 13.1.141, (Release 2022-1, Schrödinger Inc.).[S3] All structures were minimized first and the minimized structures were then used as the starting molecular structures for all MacroModel conformational searches. The force field used was MMFFs as implemented in this software ($CHCl_3$ solvation). The charges were defined by the force field library and no cut off were used for non-covalent interaction. A Polak-Ribiere Conjugate Gradient (PRCG) was used, and each structure was subjected to 10000 iterations. The minima converged on a gradient with a threshold of 0.01. Conformational search was performed from previously minimized structures using 10000 steps. Images were created using PyMol.[S4]

Calculations were performed on simplified oligomer duplexes in which the capping groups were simplified to methyl and phenyl in order to reduce the computational cost. Amidine and carboxylates were treated as charges species and the salt bridges were fixed by constraining the distance between the nitrogens and oxygens to 1.5 ± 0.5 Å. The calculation outcomes for each duplex were sorted by energy and the 25 lowest-energy conformations were analysed.

3.2. Estimation of the ring strain for the ZIP reaction

The ring strain for the ZIP reaction was estimated by using the strain energy of the product duplexes (E_{strain}). The disconnection of the triazole rings into the corresponding azide and alkyne was not used due to problems in the parameterisation of azide moieties in the MMFFs force-field. The method used for disconnecting the macrocyclic structures of the duplexes through the phenyl-triazole bonds is illustrated in Figure S1. This hypothetical transformation provides a method for calculating E_{Bond} as the energy difference between two identical fragments and the connected oligomer backbone (equation S1).

$$E_{Bond} = E_{product} - (2 \times E_{fragment}) = 467.1 - (2 \times 187.0) = \mathbf{93.0\ kJ \cdot mol^{-1}} \quad \text{(eq. S1)}$$

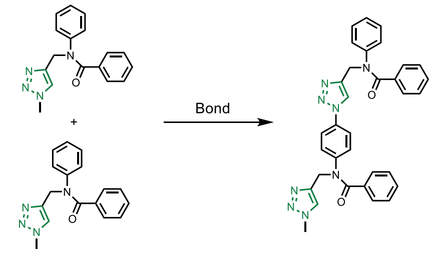

Bond fragment **Bond product**

Figure S1 Model system used to calculate bond connection energy E_{Bond} and lowest energy conformations from conformational searches using molecular mechanics (MMFFs force-field implemented in Macromodel with $CHCl_3$ solvation).[S3,S4]

The corresponding disconnection for the antiparallel and parallel dimer duplexes is illustrated in Figure S2. The energy contribution associated with phenyl-triazole bond connection (E_{bond}) was subtracted from the difference between the energy of the product duplex (E_{duplex}) and the energy of the pre-ZIP intermediate (E_{preZIP}) (equation S2).

$E_{strain} = E_{duplex} - E_{preZIP} - n\ E_{bond}$ (eq. S2)

where *n* is the number of triazole rings formed in the ZIP reaction.

The resulting ring strain is:

Antiparallel: $E_{strain} = E_{ZIP} - E_{preZIP} - E_{bond}$ = 241.2 – 127.0 – 93.0 = **21.2 kJ·mol^{-1}**

Parallel: $E_{strain} = E_{ZIP} - E_{preZIP} - E_{bond}$ = 250.7 – 127.3 – 93.0 = **30.4 kJ·mol^{-1}**

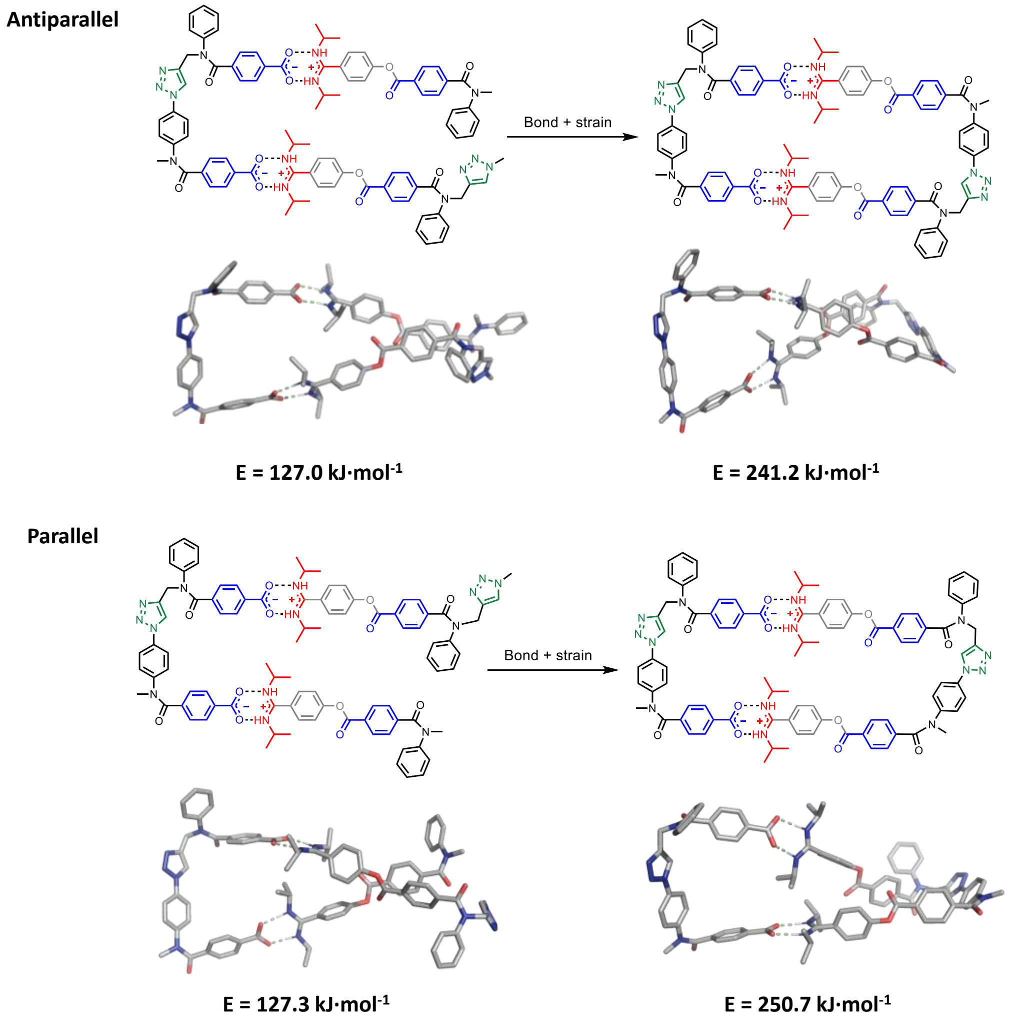

Figure S2. Calculation of ring strain for the antiparallel (A) and parallel (B) dimeric duplexes, showing the lowest energy conformations from conformational searches using molecular mechanics (MMFFs force-field implemented in Macromodel with $CHCl_3$ solvation).[S3,S4]

The ring strain for the antiparallel and parallel trimer duplexes was calculated in the same way using the disconnections illustrated in Figure S3.

Antiparallel: E_{strain} = 471.2 – 350.5 – 93.0 = **27.7 kJ·mol^{-1}**

Parallel: E_{strain} = 479.9 – 343.7 – 93.0 = **43.2 kJ·mol^{-1}**

The calculated ring strain associated with macrocyclisation in the ZIP step is 21-43 kJ·mol^{-1} per ring, which is in the order of the ring strain of common 5- and 6-membered rings.[S5-S8] These results suggest that the ring strain associated with macrocyclisation in the ZIP step is small using this backbone.

Antiparallel

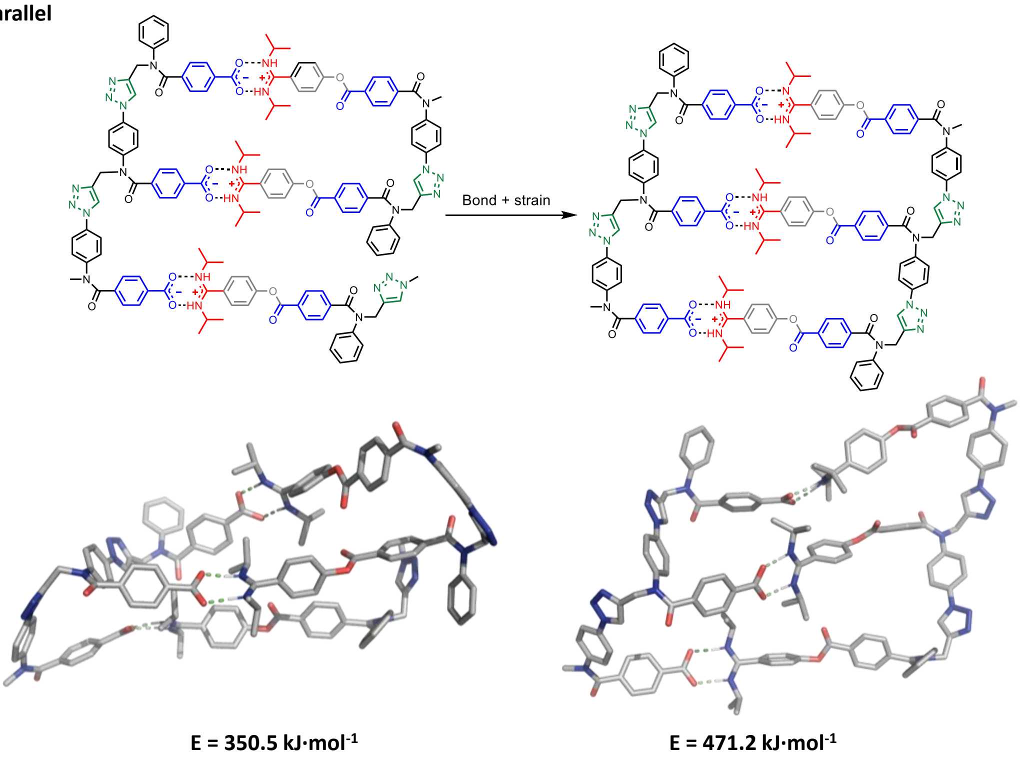

Parallel

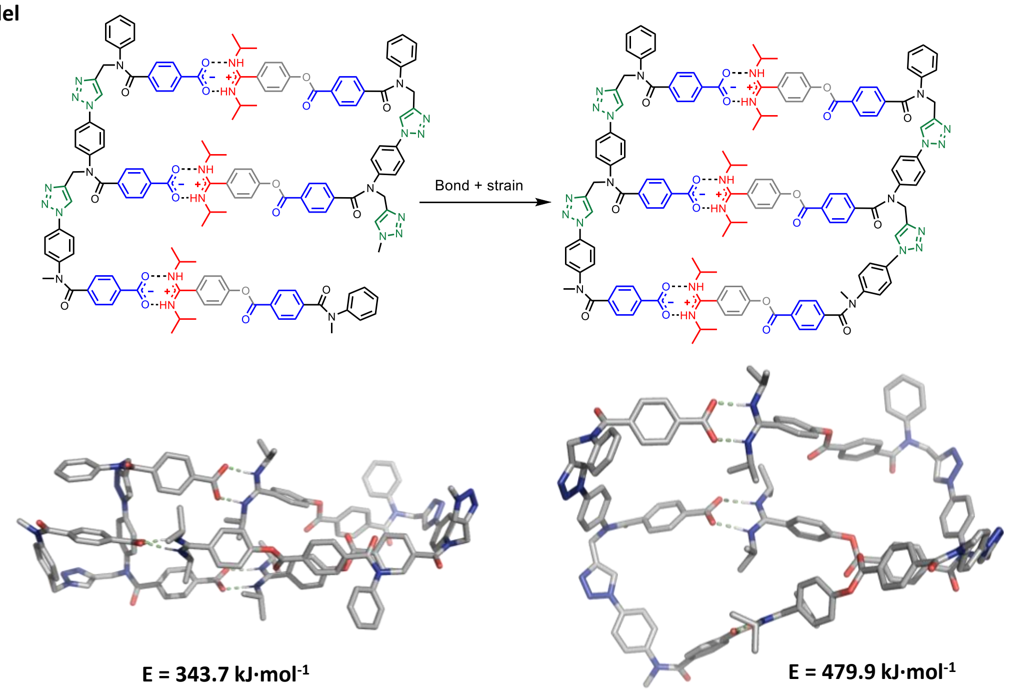

Figure S3. Calculation of ring strain for the antiparallel (A) and parallel (B) trimer duplexes showing the lowest energy conformations from conformational searches using molecular mechanics (MMFFs forcefield implemented in Macromodel with $CHCl_3$ solvation).[S3,S4]

4. Binding studies

The binding of amidine **6** to carboxylic acids under the suitable conditions for the templating experiment (mM concentration in a non-polar solvent) was assessed using ^{1}H NMR in a Bruker 500 MHz Avance III Smart Probe spectrometer. Both the free amidine monomer **6** and the salt **6·HCl** were analysed. From a 1 mL solution of monomer in $CDCl_3$ ([**6·HCl**]: 3.86 mM; [**6**]: 3.94 mM), 600 µL was added to an NMR tube. Increasing known volumes (5 µL, 5 µL, 5 µL and 20 µL) of benzoic acid were added from a 117.1 mM stock solution in $CDCl_3$ and the spectrum recorded after each addition. The chemical shifts of the **6·HCl** and **6** were monitored as a function of its concentration, in particular the amidine NH and methyl groups of the isopropyl chains. In **6**, the broad signal of the methyl groups sharpens upon addition of the guest and subsequent formation of the salt bridge. For 6·HCl, these groups are already sharp without guest and no changes in chemical shift occur upon addition of benzoic acid. Figure S4 shows the ^{1}H NMR spectra and the plot of the change in chemical shift as a function of concentration for **6·HCl** and **6**. Although the concentration of guest is too high for accurate fitting of the data, the obtained results suggest that the binding constant of amidine **6** and benzoic acid in $CDCl_3$ is bigger than 10^4 M^{-1}, in agreement with literature data,[S9-S11] while there is no interaction when the amidine is in a salt form.

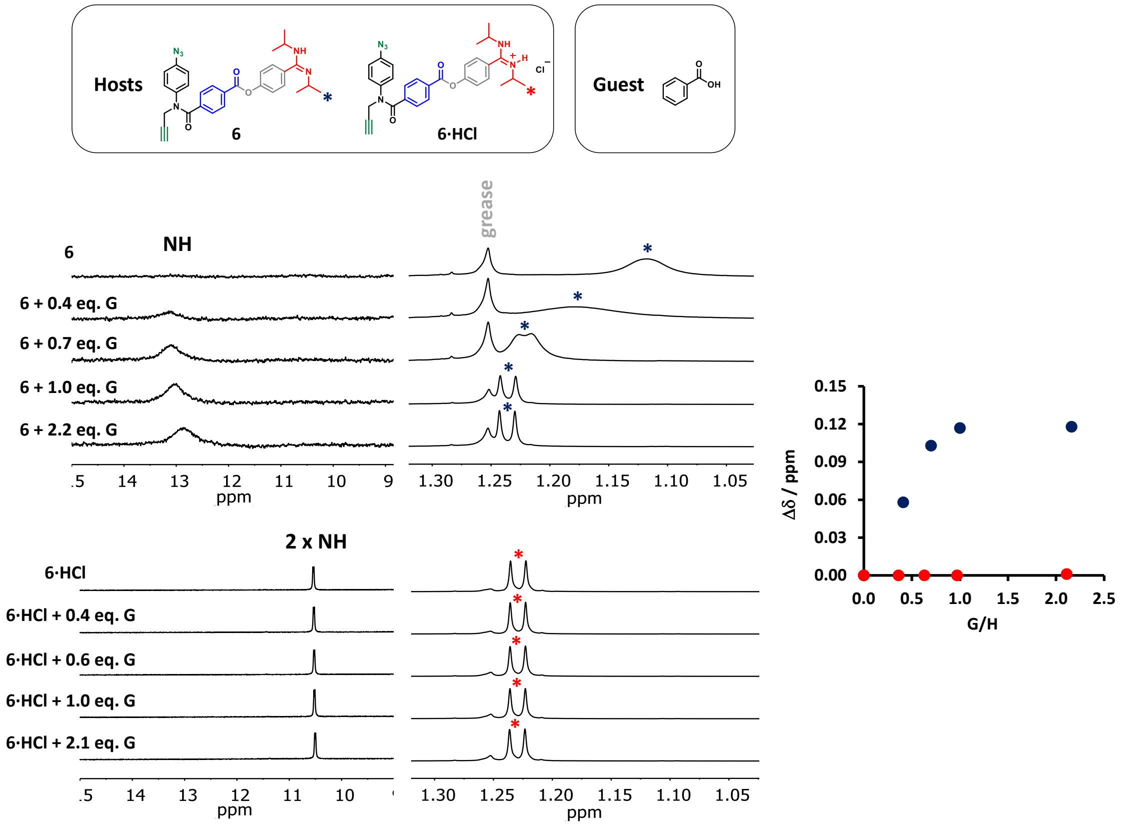

Figure S4. 500 MHz ^{1}H NMR spectra of the amidine NH and methyl groups of the isopropyl chains of the amidine in **6** and **6·HCl** for the titration of benzoic acid into **6** (3.9 mM) and **6·HCl** (3.9 mM) at 298 K in $CDCl_3$, along with the plot of the change in chemical shift of the methyl ^{1}H signal as a function of guest *vs* host ratio.

5. Template-directed synthesis

5.1. General details

The template-directed oligomerization of 6 is shown in Scheme S1. From freshly prepared stock solutions of **template** (in dry THF), **6** (in dry CH_2Cl_2) and 4-*tert*-butylbenzylazide (in CH_2Cl_2, **template** (1 eq.) and **6** (3 eq.) were mixed in a 1.75 mL vial containing a magnetic stirrer. The solvent was evaporated under N_2 stream and dry CH_2Cl_2 was added. To this solution, 4-*tert*-butylbenzylazide was added, followed by a premixed solution of $Cu(CH_3CN)_4PF_6$ (6 eq.) and TBTA (6 eq.) in dry CH_2Cl_2 (10 µL). The vial was flushed briefly with N_2, sealed and left stirring at room temperature for 2 days. Two aliquots were taken after this time, one for UPLC analysis and the second for cleavage. Both aliquots were evaporated to remove volatile 4-tert-butylbenzylazide. One of them was dissolved in CH_3CN/H20 2:1 and analyzed by UPLC. The second was dissolved in THF:H_2O (3:1, 1mL) and two drops of 1M LiOH aq. soln. were then added. The solution was left to react for 1h and then analyzed by UPLC. The same procedure was followed for the non-templated oligomerization shown in Scheme S2, but no template was added.

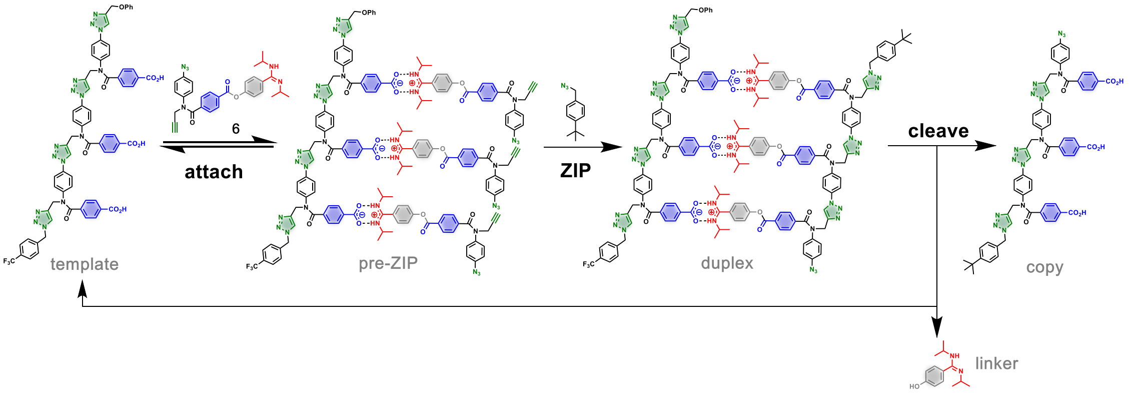

Scheme S1. CuAAC templated oligomerization of **6.**

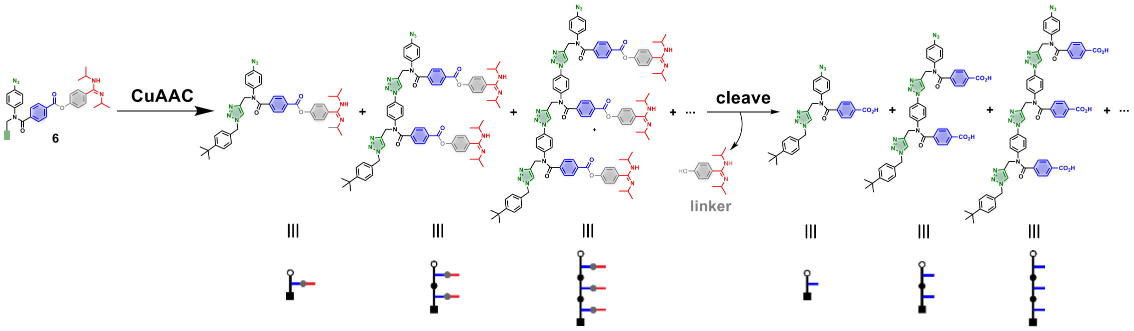

Scheme S2. CuAAC non-templated oligomerization of **6.**

5.2. Template concentration dependency on templating

Initially, the effect of the concentration of template and monomer **6** in the templating process was analysed. For that purpose, three different conditions were tested with [**template**] of 0.01, 0.1 and 1 mM and 3 equivalents of **6** in each case (Figure S5). In all the cases, the concentration of capping azide (4-*tert*-butylbenzylazide) used was 1 mM. Figure S6 shows the MS spectra for the template and obtained oligomers. Figure S7 shows the UPLC

traces corresponding to the hydrolysis of the oligomerization crude reaction mixtures while MS spectra of the obtained species is provided in Figure S8.

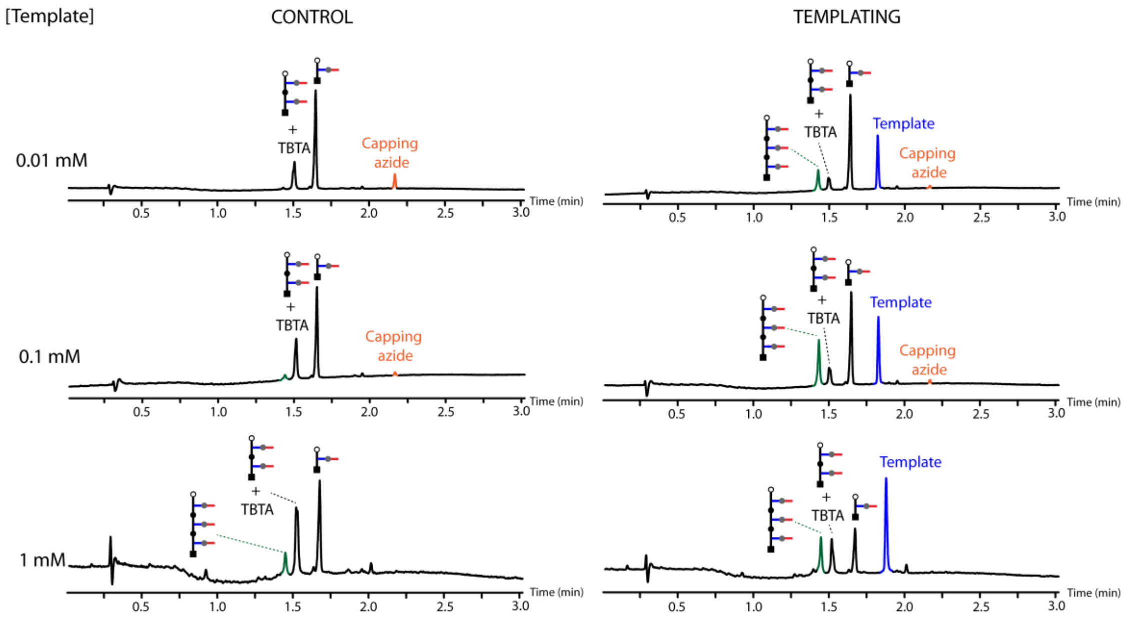

Figure S5. Control and templating experiments highlighted in Scheme S1 at 0.01, 0.1 and 1 mM of **template** with [*tert*-butylbenzylazide] of 1 mM, 3 eq. of **6** and 6 eq. of Cu-TBTA. In the control experiment, only 1-mer **6**, capping azide and Cu-TBTA were used.

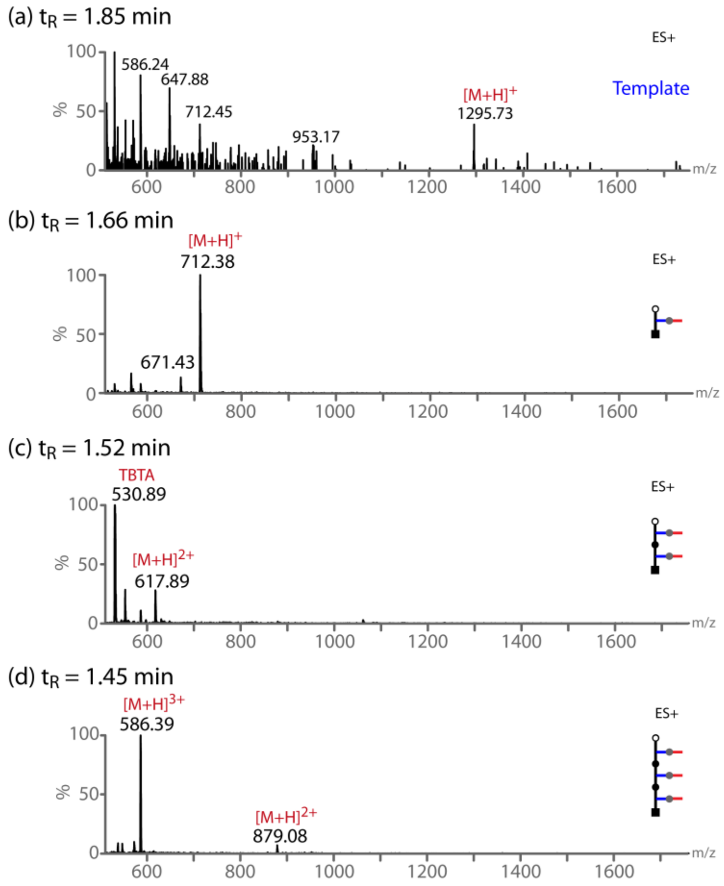

Figure S6. MS spectra of template and obtained oligomers (MW: template 1293.4, 1-mer 711.4, 2-mer 1233.6, 3-mer 1755.8).

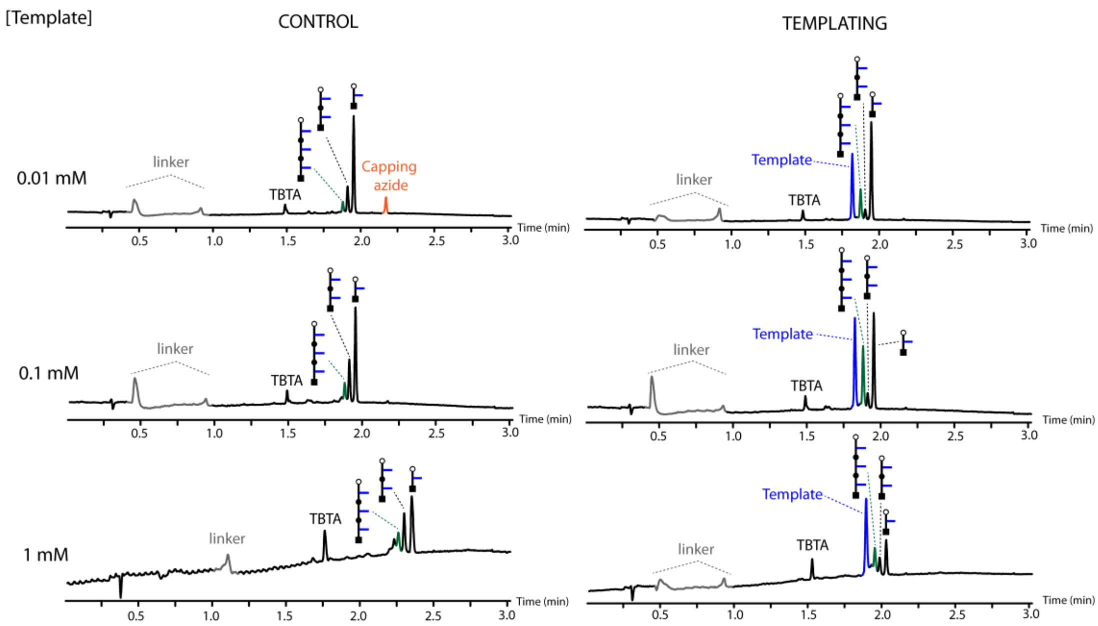

Figure S7. Hydrolysis of the oligomerization crude reaction mixtures shown in Figure S5.

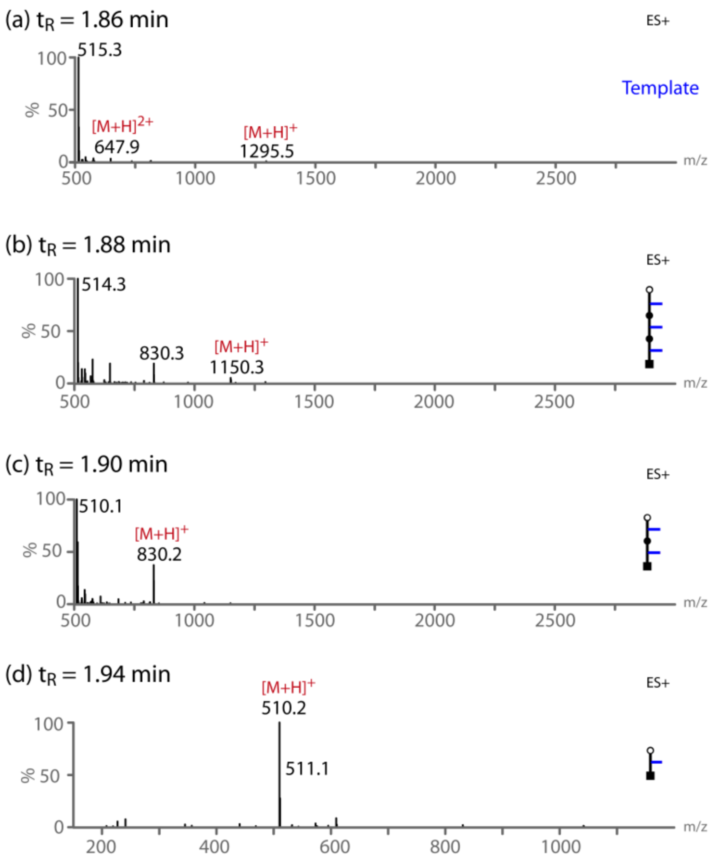

Figure S8. MS spectra of template and obtained oligomers after hydrolysis (MW: template 1293.4, 1-mer 509.2, 2-mer 829.3, 3-mer 1149.4).

The output of the templated and control oligomerization reactions was assessed by UPLC. The ratio of the areas at 254 nm for the 2-mer (off-template pathway) over the 3-mer (templated) oligomer was calculated in each case (χ = 2-mer/3-mer). The ratio of these vales for the templating (χ_{temp}) over the control ($\chi_{control}$) experiment (χ_{temp} / $\chi_{control}$) was plotted as a function of the concentration of **template** (Figure S9). Optimal templating is obtained when a 0.01 and 0.1 mM concentration of template is used, while more concentrated reaction leads to a loss in the template effect as the off-template pathway is favoured (more off-template 2-mer oligomer and less templated 3-mer oligomer are formed).

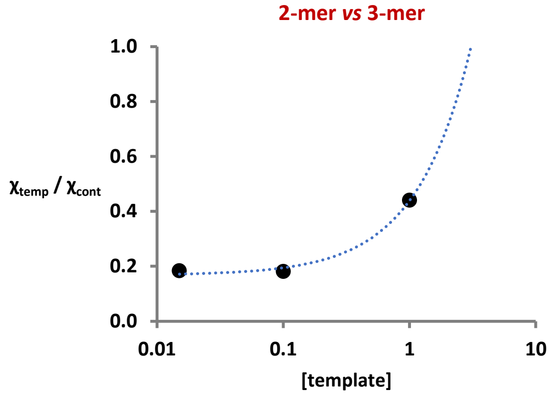

Figure S9. Plot of the ratio of 2-mer *vs* 3-mer oligomers between templating and control experiments as a function of the concentration of template ([capping azide] = 1 mM). The line represents the best fit of the data to χ_{temp} / $\chi_{control}$ = 0.27 [**template**] + 0.17 (R^2 = 0.99).

5.3. Solvent dependency on templating

Two different solvents were screened in order to find the most suitable one for promoting the templating reaction. Amidine-carboxylate has been reported to be stable over a wide range of non-polar solvents.[S9-S11] However, in this case, the change of CH_2Cl_2 for THF led to a complete loss of the template effect. As shown in Figure S10, when THF is used, both control and templating experiment UPLC traces are similar, with no preferential formation of 3-mer oligomer when the template is present in the reaction.

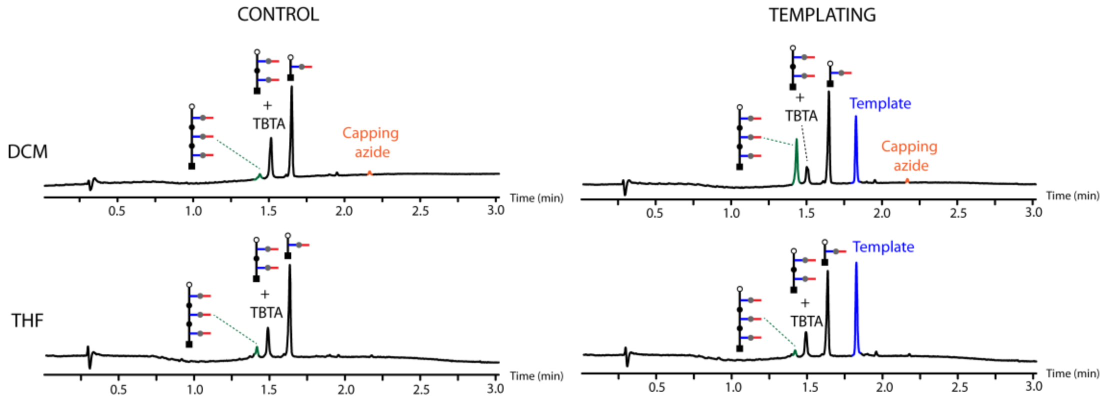

Figure S10. Control and templating experiments highlighted in Scheme 2 with 0.1 mM of template and [capping azide] of 1 mM, 3 eq. of **6** and 6 eq. of Cu-TBTA, in DCM and THF. In the control experiment, only 1-mer **6**, capping azide and Cu-TBTA were used.

5.4. Cap concentration dependency on templating

We have previously reported the relevance of the cap concentration on the success of covalent template-directed synthesis of linear oligomer.[S12,S13] We screened the concentration of cap from 0 to 1 mM, using the best conditions from previous experiments (0.1 mM of template, 0.3 mM of 6 in CH_2Cl_2) in order to find the optimal cap concentration. This in turn allows the estimation of the effective molarity for the ZIP process. Figure S11 shows the UPLC traces for control and templating experiments with variable cap concentration. Figure S12 shows the UPLC traces corresponding to the hydrolysis of the oligomerization crude reaction mixtures.

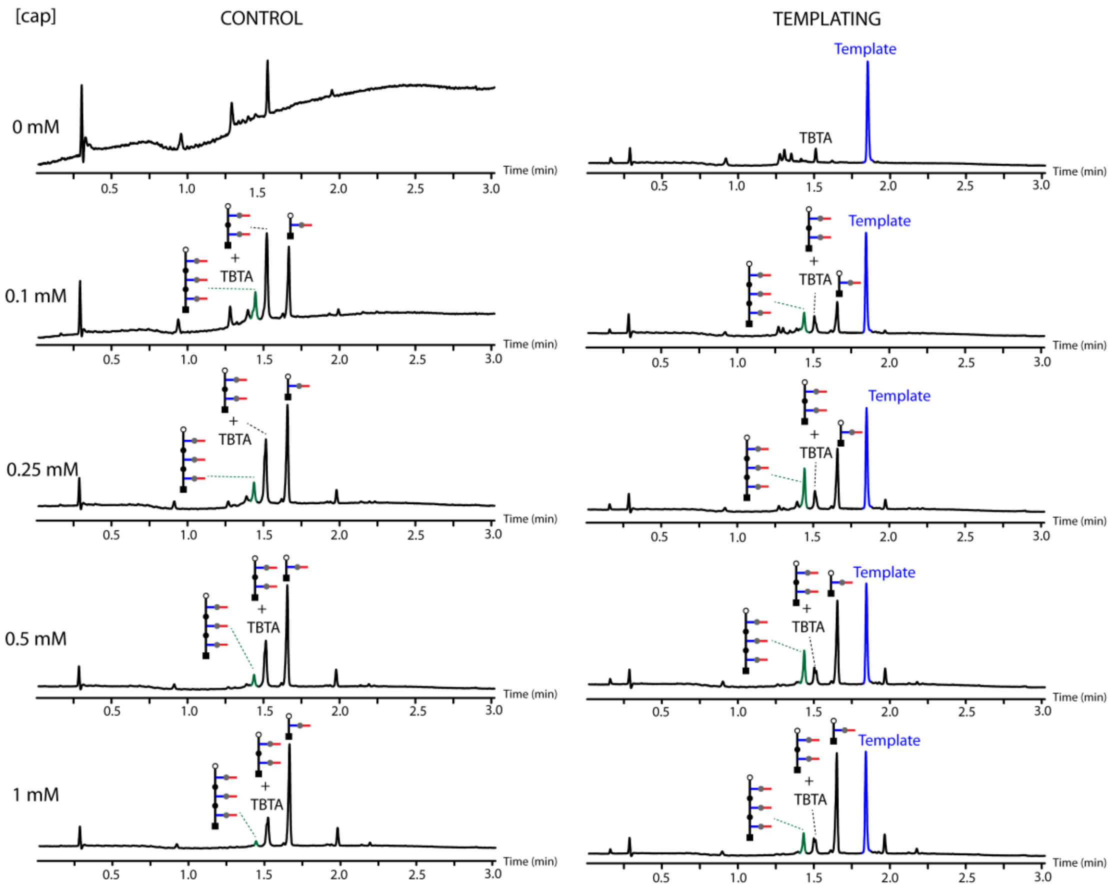

Figure S11. Control and templating experiments highlighted in Scheme S1 with 0.1 mM of template, variable concentration of capping azide, 3 eq. of **6** and 6 eq. of Cu-TBTA. In the control experiment, only 1-mer **6**, capping azide and Cu-TBTA were used.

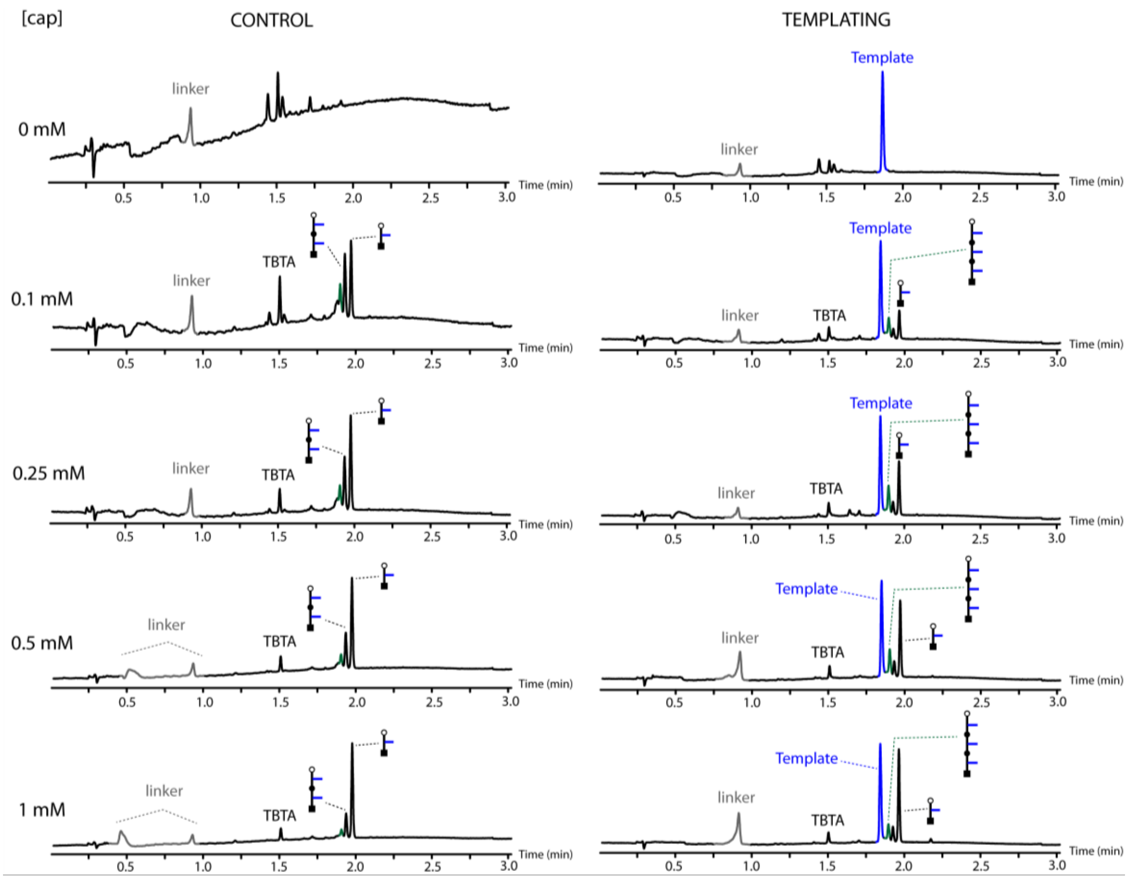

Figure S12. Hydrolysis of the oligomerization crude reaction mixtures shown in Figure S11.

Again, the output of the templated and control oligomerization reactions was assessed by UPLC. χ_{temp} / $\chi_{control}$ was plotted as a function of the concentration of end-capping agent (Figure S13), which shows how the product distribution depends on the concentration of the capping agent.

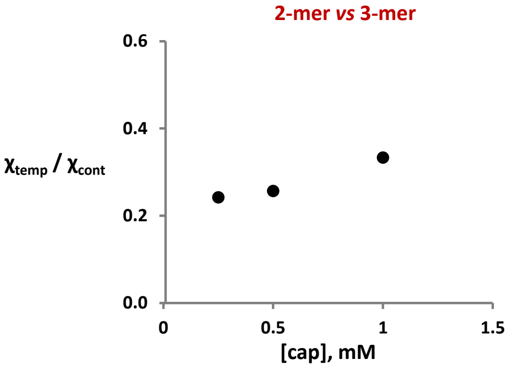

Figure S13. Plot of the ratio of 2-mer vs 3-mer oligomers between templating and control experiments as a function of the concentration of capping azide ([**template**] = 0.1 mM). For the estimation of the EM, only the last three data points (right graph) were fitted to χ_{temp} / $\chi_{control}$ = 0.13 [**cap**] + 0.20 (R^2 = 0.97).

The ratio of the rates of off-template reaction (monitored via the formation of 2-mer oligomer) and intramolecular reaction (monitored via the formation of templated 3-mer

oligomer) is expected to be a linear function of the concentration of the capping agent (equation S3):

$$A_{off\text{-}template}/A_{templating} = c\,[cap] \qquad \text{(eq. S3)}$$

The data in Figure S11 can be used to determine values of effective molarity (EM) for the ZIP process by extrapolating the curve to the concentration of capping agent necessary to obtain a 1:1 ratio of the two products. These concentrations were corrected for the 5-fold difference in reactivity measured for the aromatic and aliphatic azide.[S12,S13] The value of EM estimated is 32 mM.

6. References

[S1] D. Núñez-Villanueva, C. A. Hunter. Molecular replication using covalent base-pairs with traceless linkers. *Org. Biomol. Chem.* **2019**, *17*, 9660.

[S2] D. Núñez-Villanueva, M. Ciaccia, G. Iadevaia, E. Sanna, C. A. Hunter. Sequence information transfer using covalent template-directed synthesis. *Chem. Sci*. **2019**, *10*, 5258.

[S3] Schrödinger Release 2022-1: MacroModel, Schrödinger, LLC, New York, NY, 2022 (version 13.1.141).

[S4] The PyMOL Molecular Graphics System (open-source PyMOL). Version 2.3.0a0. Schrödinger, LLC.

[S5] T. Dudev, C. Lim. Ring strain energies from *ab initio* calculations. *J. Am. Chem. Soc.* **1998**, *120*, 4450.

[S6] W. Saiyasombat, R. Molloy, T. M. Nicholson, A. F. Johnson, I. M. Ward, S. Poshyachinda. Ring strain and polymerizability of cyclic esters. *Polymer*, **1998**, *39*, 5581.

[S7] P. R. Khoury, J. D. Goddard, W. Tam. Ring strain energies: substituted rings, norbornanes, norbornenes and norbornadienes. *Tetrahedron* **2004**, *60*, 8103.

[S8] A Hejl, O. A. Scherman, R. H. Grubbs. Ring-opening metathesis polymerization of functionalized low-strain monomers with ruthenium-based catalysts. *Macromolecules* **2005**, *38*, 7214.

[S9] Y. Tanaka, H. Katagiri, Y. Furusho, E. Yashima. A modular strategy to artificial double helices. *Angew. Chem. Int. Ed.* **2005**, *44*, 3867.

[S10] H. Yamada, Z.-Q. Wu, Y. Furusho, E. Yashima. Thermodynamic and kinetic stabilities of complementary double helices utilizing amidinium–carboxylate salt bridges. *J. Am. Chem. Soc.* **2012**, *134*, 9506.

[S11] X. Ren, X. Wang, Y. Sun, X. Chi, D. Mangel, H. Wang, J. L. Sessler. Amidinium–carboxylate salt bridge mediated proton-coupled electron transfer in a donor–acceptor supramolecular system. *Org. Chem. Front.* **2019**, *6*, 584.

[S12] M. Ciaccia, D. Núñez-Villanueva, C. A. Hunter. Capping strategies for covalent template-directed synthesis of linear oligomers using CuAAC. *J. Am. Chem. Soc.* **2019**, *141*, 10862.

[S13] D. Núñez-Villanueva, M. Ciaccia, C. A. Hunter. Cap control: cyclic versus linear oligomerisation in covalent template-directed synthesis. *RSC Adv*. **2019**, *9*, 29566.
